# Inflammation-induced mitochondrial and metabolic disturbances in sensory neurons control the switch from acute to chronic pain

**DOI:** 10.1016/j.xcrm.2023.101265

**Published:** 2023-11-08

**Authors:** Hanneke L.D.M. Willemen, Patrícia Silva Santos Ribeiro, Melissa Broeks, Nils Meijer, Sabine Versteeg, Annefien Tiggeler, Teun P. de Boer, Jędrzej M. Małecki, Pål Ø. Falnes, Judith Jans, Niels Eijkelkamp

**Affiliations:** 1 Center for Translational Immunology, Department of Immunology, University Medical Center Utrecht, Utrecht University, 3508 Utrecht, the Netherlands; 2 Section Metabolic Diagnostics, Department of Genetics, University Medical Center Utrecht, Utrecht University, 3508 Utrecht, the Netherlands; 3 Department of Medical Physiology, Division of Heart & Lungs, University Medical Center Utrecht, Yalelaan 50, 3584 Utrecht, the Netherlands; 4 Department of Biosciences, Faculty of Mathematics and Natural Sciences, University of Oslo, Oslo, Norway; 5 CRES–O - Centre for Embryology and Healthy Development, University of Oslo and Oslo University Hospital, Oslo, Norway

**Keywords:** mitochondria, metabolism, redox, sensory neurons, inflammation, chronic pain

## Abstract

Pain often persists in patients with an inflammatory disease, even when inflammation has subsided. The molecular mechanisms leading to this failure in pain resolution and the transition to chronic pain are poorly understood. Mitochondrial dysfunction in sensory neurons links to chronic pain, but its role in resolution of inflammatory pain is unclear. Transient inflammation causes neuronal plasticity, called hyperalgesic priming, which impairs resolution of pain induced by a subsequent inflammatory stimulus. We identify that hyperalgesic priming in mice increases the expression of a mitochondrial protein (ATPSc-KMT) and causes mitochondrial and metabolic disturbances in sensory neurons. Inhibition of mitochondrial respiration, knockdown of *ATPSCKMT* expression, or supplementation of the affected metabolite is sufficient to restore resolution of inflammatory pain and prevents chronic pain development. Thus, inflammation-induced mitochondrial-dependent disturbances in sensory neurons predispose to a failure in resolution of inflammatory pain and development of chronic pain.

## Highlights

• Mitochondrial and metabolic disturbances persist in sensory neurons after inflammation
• Disturbed redox balance in DRG predisposes to failing inflammatory pain resolution
• Mitochondrial ATPSc-KMT links to disturbed cellular redox balance
• *ATPSCKMT* knockdown or NAD^+^ supplementation restores failing pain resolution pathways

## Graphical abstract

**Figure:**
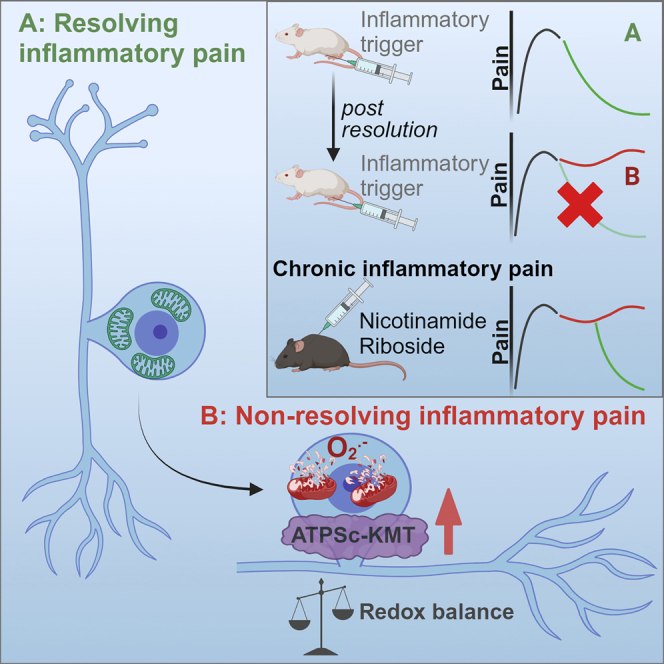

## Teaser

Willemen et al. identified that transient inflammatory pain causes mitochondrial and redox changes in sensory neurons that persist beyond resolution. These changes predispose to a failure in resolution of pain caused by a subsequent inflammation. Additionally, targeting the cellular redox balance prevents and treats chronic inflammatory pain in rodents.

## Introduction

Chronic pain is a leading cause of years lived in disability, yet treatment options are limited and often induce severe side effects.^1^^,^^2^ The current dogma is that pain resolution is the consequence of the dissipation of the drivers that induced the pain. However, in 12%–30% of patients with rheumatic arthritis, pain persists while they have minimal joint inflammation or are in remission.^3^^,^^4^^,^^5^ Accumulating evidence indicates that pain resolution after tissue damage or inflammation is not a passive but rather an active process that involves endogenous pain resolution mechanisms.^6^^,^^7^^,^^8^ Failed pain resolution pathways may lead to the transition from acute to chronic pain. However, the molecular mechanisms that contribute to failure in pain resolution are not well understood.

Mitochondria play a crucial role in maintaining neuronal homeostasis by ensuring metabolic functions and energy production in the form of adenosine triphosphate (ATP) via oxidative phosphorylation (OXPHOS).^9^^,^^10^ Moreover, mitochondria are essential to regulate multiple cellular processes, such as calcium homeostasis, ion channel activity, and reactive oxygen species (ROS) signaling.^9^^,^^10^ Deficits in mitochondrial functions are linked to chronic pain. In humans, approximately 70% of the patients with heritable mitochondrial diseases have chronic pain.^11^ A genetic polymorphism in the mitochondrial 16S rRNA gene (gene MT-RNR2) increases the risk of developing fibromyalgia.^12^ Similarly, several preclinical studies have linked mitochondrial dysfunction (e.g., reduced ATP production) in sensory neurons to chronic pain in rodents, although predominantly in chemotherapy-induced chronic pain models.^13^^,^^14^^,^^15^ In addition, the mitochondrial protein ATPSc-KMT (formerly named FAM173B), a lysine (K)-specific methyltransferase (MTase), influences OXPHOS activity by methylating Lys-43 of the ATP synthase c-subunit (ATPSc) and promotes chronic pain development.^16^^,^^17^ Finally, OXPHOS in sensory neurons adapts during transient inflammatory pain, and donation of mitochondria from macrophages to sensory neurons is needed to resolve inflammatory pain.^7^ Thus, we hypothesize that adequate regulation of mitochondrial activity in sensory neurons is required for resolution of inflammatory pain. We here set out to better understand mechanistically how mitochondria in sensory neurons are involved in pain resolution or its failure, leading to the transition from acute to chronic pain.

It is well known that a peripheral inflammation induces long-lasting molecular changes in sensory neurons, a phenomenon called hyperalgesic priming.^18^^,^^19^ These changes promote chronic pain development after a subsequent inflammatory insult, which normally causes only transient hyperalgesia in non-primed mice.^18^^,^^19^ Thus, a priming model may not only help to identify mechanisms that promote chronic pain but can also be viewed as a model that causes impairments in endogenous pain resolution mechanisms. Here, we tested whether transient inflammation causes long-lasting disturbances in mitochondrial and metabolic activity in sensory neurons and whether these are at the core of failure in resolution of inflammatory pain and development of chronic inflammatory pain.

## Results

### Transient inflammation causes persistent changes in mitochondrial respiration in the soma of sensory neurons

**Figure 1:**
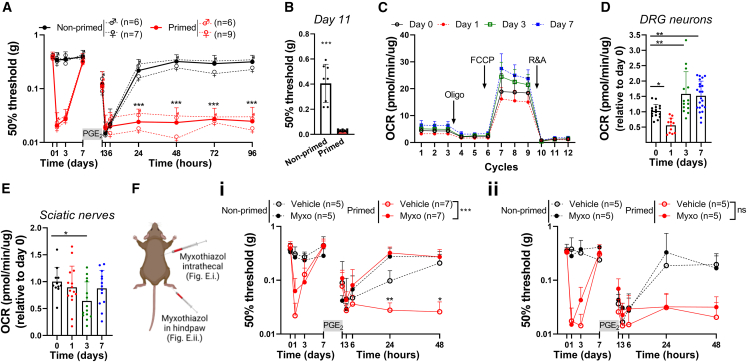
Increased mitochondrial activity in DRG neurons impairs resolution of PGE_2_-induced hyperalgesia in primed mice (A) Course of mechanical hyperalgesia in male (**♂**) and female (♀) mice after intraplantar injection of carrageenan (1% w/v, 5 μL, primed) or vehicle (non-primed). At day 7, mice received a subsequent intraplantar injection with PGE_2_ (100 ng/paw). (B) Mechanical hypersensitivity at day 11 after PGE_2_ (which is day 18 after carrageenan) in primed and non-primed mice. (C) OCR in DRG neuron cultures at indicated days after intraplantar carrageenan injection. OCR was measured under basal conditions and after sequential addition of oligomycin (oligo; ATP synthase inhibitor), carbonyl cyanide-p-trifluoromethoxyphenylhydrazone (FCCP; uncoupling protonophore that dissipates mitochondrial membrane potential), and a mixture of rotenone (inhibitor of complex I) and antimycin A (inhibitor of complex III) (R&A). (D) Basal respiration of DRG neurons at days 0 (n = 16), 1 (n = 12), 3 (n = 14), and 7 (n = 22) after intraplantar carrageenan injection. Each dot represents the respiration measured in a well with sensory neurons. Lumbar (L3–L5) DRGs from one or two mice were pooled per experiment, divided over 3–5 wells and assessed in 3 experiments. (E) Basal respiration of sciatic nerves at days 0 (n = 11), 1 (n = 15), 3 (n = 14), and 7 (n = 13) after intraplantar carrageenan injection. Each dot corresponds to one sciatic nerve innervating an injected paw. (F) Course of PGE_2_-induced mechanical hyperalgesia after (i) i.t. or (ii) intraplantar injection of vehicle or myxothiazol (myxo; 50 μM) at day 7 (15 min prior to intraplantar PGE_2_) in carrageenan-primed and non-primed mice. Data are represented as mean ± SD. ∗p < 0.05, ∗∗p < 0.01, ∗∗∗p < 0.001. Statistical analyses were performed by Student’s t test (B), one-way ANOVA (D and E) followed by Dunnett’s multiple comparison test, or two-way repeated measures ANOVA followed by a post hoc Sidak’s multiple comparison test (A and F; stars indicate significance comparing primed conditions; NS, not significant).

Carrageenan is a well-known priming stimulus that induces transient inflammatory hyperalgesia and programs sensory neurons to respond differently to a subsequent inflammatory stimulus after carrageenan-induced hyperalgesia has resolved.^18^ Injection of carrageenan into the hind paw (intraplantar) of mice induced transient inflammatory mechanical hyperalgesia, as assessed with the von Frey test. Hyperalgesia peaked at day 1 and resolved within 3–4 days (Figure 1A). At day 7, when mechanical hyperalgesia had completely resolved, mice received an intraplantar injection of prostaglandin E2 (PGE_2_) to unmask the primed state. PGE_2_-induced hyperalgesia persisted in primed male and female mice that did not resolve for at least 11 days. In contrast, in non-primed vehicle-injected mice, PGE_2_-induced hyperalgesia resolved within 1 day (Figures 1A and 1B). Since the course of mechanical hyperalgesia was not significantly different between males and females, mice of both sexes were used in subsequent experiments. To investigate if the presence of hyperalgesic priming at day 7 is concurrent with mitochondrial adaptations in dorsal root ganglia (DRGs), which contain the soma of sensory neurons innervating the inflamed paw, we measured oxygen consumption rates (OCRs) *ex vivo* as a measure of mitochondrial respiration. At the peak of inflammatory pain (day 1), basal mitochondrial respiration was reduced compared with baseline but increased again when inflammatory hyperalgesia started resolving (at day 3; Figures 1A–1D), consistent with previous findings.^7^ Surprisingly, at day 7, when mechanical sensitivity had returned to baseline (Figure 1A), basal mitochondrial respiration was significantly increased compared with the respiratory activity at day 0 (Figure 1D). Dissection of basal mitochondrial respiration into three other separate key mitochondrial respiration parameters, using pharmacological mitochondrial-complex-specific inhibitors, showed that respiration due to proton leak, ATP-synthesis-linked respiration, and maximal respiration were all increased at day 7 compared to mice without previous inflammation (Figure S1A). Neither mitochondrial mass nor the expression of OXPHOS complexes (I–V) in the lumbar DRG of primed mice were significantly affected (Figures S1B–S1E). Moreover, at day 7 after hyperalgesic priming, no signs of ongoing paw inflammation were detected, since mRNA expression of pro-inflammatory cytokines (*IL1-β*, *IL-6*) and a macrophage marker (*F4/80*) were similar to non-primed mice (Figure S1F). These data suggest that hyperalgesic priming causes increased mitochondrial respiration in DRG neurons, without clear persisting paw inflammation.

Since energy demand may differ between the sensory neuron’s cell body and its axons, we also determined mitochondrial respiration in the sciatic nerves during the course of carrageenan-induced hyperalgesia. During the peak of inflammatory hyperalgesia (day 1), basal mitochondrial respiration was similar to baseline (Figures 1E and S1G). At day 3, basal mitochondrial respiration was decreased but recovered again at day 7 to similar levels as at day 0 (Figure 1E). A similar trend was observed for proton leak, ATP-synthesis-linked respiration, and maximal respiration (Figure S1H). These data indicate that after recovery from carrageenan-induced hyperalgesia, mitochondrial respiratory activity is selectively increased in the soma of sensory neurons innervating the injected paws.

### Enhanced respiratory activity in DRG neurons is linked to failure in the resolution of PGE_2_-induced hyperalgesia

We tested whether the increase in mitochondrial respiratory activity in DRG neurons contributes to the failure to resolve PGE_2_-induced hyperalgesia in primed mice. To decrease mitochondrial respiration in the lumbar DRG, mice received an intrathecal injection with myxothiazol (inhibitor of complex III of the electron transport chain [ETC]).^20^ This administration route is effective to deliver drugs to DRG neurons and significantly reduced mitochondrial respiration in DRG neurons of myxothiazol-treated mice (Figure S1I).^21^ Intrathecal administration of myxothiazol prior to PGE_2_ injection completely restored the resolution of PGE_2_-induced mechanical and thermal hyperalgesia in carrageenan-primed mice (Figures 1Fi and S1J). In contrast, intraplantar injection of myxothiazol, to target nerve endings or other (local) cells, prior to PGE_2_ injection did not restore resolution of PGE_2_-induced hyperalgesia in primed mice (Figure 1Fii). These data indicate that the enhanced mitochondrial respiratory activity in the soma of DRG neurons, but not in the nerve endings, contributes to the carrageenan-induced hyperalgesic priming state and the associated failure in resolution of hyperalgesia, induced by a subsequent inflammatory trigger.

### Hyperalgesic priming induces disturbances in redox balance and oxidative stress in DRG

**Figure 2:**
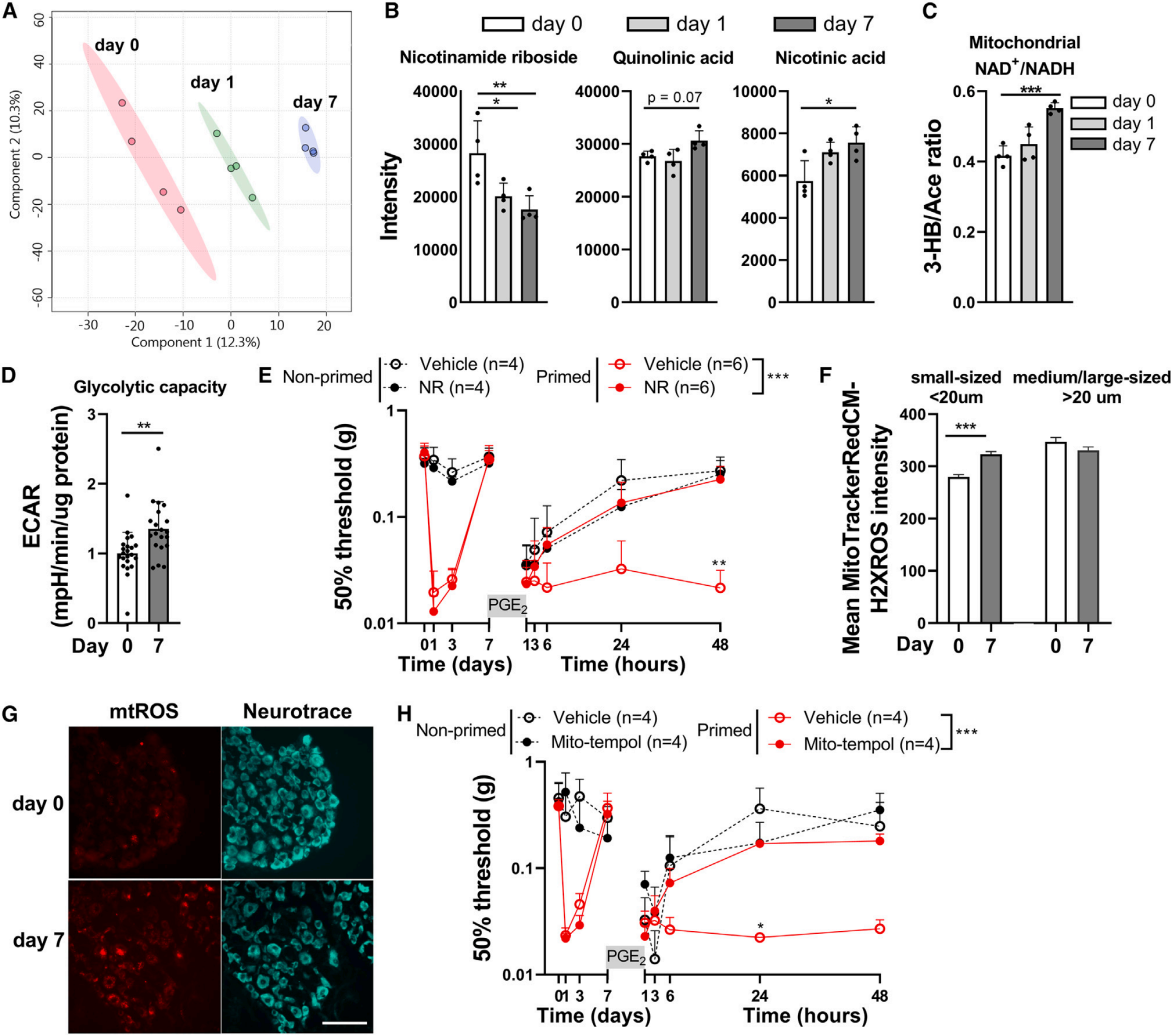
NAD^+^ supplementation and attenuation of oxidative stress restores resolution of PGE_2_-induced hyperalgesia in primed mice (A) Supervised partial least squares discriminant analysis of DI-HRMS data from lumbar DRGs isolated from naive mice (day 0) and mice treated with intraplantar carrageenan (1 or 7 days post-treatment). Each dot represents a metabolite signature of the lumbar DRG isolated from one mouse. (B and C) Intensity of metabolites measured by DI-HRMS (B) involved in generation of NAD^+^ (C) or used to measure 3-hydroxybutyrate/acetoacetate ratio as an indirect measure of mitochondrial NAD^+^/NADH ratio (n = 4). (D) Extracellular acidification rate (ECAR) in cultured DRG neurons from naive mice (non-primed, n = 22) or mice that had resolved from carrageenan-induced inflammatory hyperalgesia (primed, day 7, n = 20). (E) Course of PGE_2_-induced mechanical hyperalgesia after intraperitoneal injection with nicotinamide riboside (NR; 500 mg/kg) at day 7 in carrageenan-primed or non-primed mice and 15 min prior to intraplantar injection of PGE_2_. (F and G) Detection of mtROS formation in DRG neurons of naive mice (day 0) or mice that had resolved from carrageenan-induced inflammatory hyperalgesia (day 7). mtROS was visualized by an i.t. injection with MitoTrackerRedCM-H2XROS (100 μM), which accumulates in mitochondria and generates fluorescence upon oxidation by mtROS. (F) Mean MitoTrackerRedCM-H2XROS fluorescence intensity in small- and medium-/large-diameter neurons at indicated days (small diameter n = 700–1,000 cells, medium/large sized = 250–550 cells). (G) Representative pictures of (F). Neurons are visualized with NeuroTrace (blue; scale bar: 100 μM). (H) Course of PGE_2_-induced mechanical hyperalgesia after i.t. administration of Mito-TEMPOL (25 μg) at day 7 in carrageenan-primed or non-primed mice and 15 min prior to intraplantar injection of PGE_2_. Data are represented as mean ± SD. ∗p < 0.05, ∗∗p < 0.01, ∗∗∗p < 0.001. Statistical analyses were performed by Student’s t test (D), one-way ANOVA (B, C, and F) followed by Dunnett’s multiple comparison test, or two-way repeated measures ANOVA followed by a post hoc Sidak’s multiple comparison test (E and H; stars indicate significance comparing primed conditions).

Since changes in mitochondrial respiration may affect cellular metabolism,^22^ we tested whether the metabolic state in the lumbar DRG is affected during peripheral inflammation and after its resolution, at a time point when mice are primed. To that end, we performed a direct-infusion high-resolution mass spectrometry (DI-HRMS) on lumbar DRG lysates at various time points during carrageenan-induced hyperalgesia. With this method, we detected ∼1,900 mass peaks corresponding to ∼3,800 metabolites (including isomers). We performed a supervised partial least squares discriminant analysis to acquire distinct metabolic profiles (Figure 2A). Subsequently, we investigated which specific metabolites are important in making those distinct profiles, followed by a metabolic pathway analysis to predict which pathways are affected by hyperalgesic priming. Explorative pathway analysis of metabolites detected in the lumbar DRG at baseline (day 0) versus the DRG of primed mice (day 7 after carrageenan) suggests that metabolites involved in ubiquinone synthesis, vitamin B6 metabolism, and nicotinamide metabolism are mostly affected when mice had recovered from carrageenan-induced hyperalgesia but were primed (Figure S2A). We verified these findings by looking at the raw intensity data of metabolites involved in these pathways at days 0, 1, and 7 after carrageenan injection of the same dataset, since data scaling and outliers may influence the outcome of the multivariate analysis. Intensities of metabolites assigned to nicotinamide metabolism, but not to ubiquinone synthesis and vitamin B6 metabolism, were significantly changed after resolution of carrageenan-induced hyperalgesia (Figures 2B and S2B). These include nicotinic acid, nicotinamide riboside (NR), and quinolinic acid, which are linked to NAD^+^ biosynthesis via the Preiss-Handler pathway, salvage pathway, or *de novo* synthesis via L-tryptophan (Trp), respectively. NAD^+^ has emerged as an essential cofactor regulating mitochondrial fitness and many redox reactions.^23^ Compared to day 0 (naive mice), nicotinic acid levels were significantly increased at day 7, when mice had recovered from carrageenan-induced hyperalgesia, but not during the peak of inflammatory pain (day 1). Quinolinic acid levels were also slightly increased at day 7 but not significantly compared to DRGs isolated from naive mice (day 0, p = 0.07). In contrast, NR was significantly reduced at days 1 and 7 after carrageenan compared to naive mice (Figure 2B). In an additional independent experiment, NR intensity levels were significantly reduced in DRGs of primed mice that had resolved from inflammatory pain (7 days after carrageenan) compared to the peak of inflammatory pain (day 1) (Figure S2C), validating these findings. These results indicate that after resolution of a transient peripheral inflammation, when sensory neurons are primed for subsequent inflammatory triggers, formation of NAD^+^ precursors is affected in DRG neurons and/or non-neuronal cells, such as immune and glia cells.

NAD^+^ and its reduced form, NADH, are mainly found in three cellular pools: cytosol, nucleus, and mitochondria. Cytosolic and nuclear NAD^+^ concentrations are typically similar, as NAD^+^ and NADH move freely through pores in the nuclear membrane.^24^ However, mitochondrial NAD^+^ concentrations can be different from cytosolic and nuclear NAD^+^ concentrations because the mitochondrial inner membrane is impermeable to NADH.^24^ To investigate how the mitochondrial NAD^+^/NADH redox balance was affected in the DRG after resolution of inflammatory pain in primed mice, we measured the 3-hydroxybutyrate/acetoacetate ratio as a proxy for the mitochondrial NAD^+^/NADH ratio.^25^ At day 7 after intraplantar carrageenan administration, the 3-hydroxybutyrate/acetoacetate ratio was increased compared to day 0 (Figure 2C). These data suggest an increase in the mitochondrial NAD^+^/NADH ratio, either due to higher mitochondrial NAD^+^ or lower mitochondrial NADH concentrations. The latter could be caused by reduced NADH generation in the tricarboxylic acid (TCA) cycle or increased NADH consumption by complex I to support increased OXPHOS activity, which we observed in primed mice (Figures 1C and 1D).

In the cytosol, the NAD^+^/NADH redox state is strongly determined by glycolysis, which promotes the balance toward increased NADH levels.^26^ The ratio between the glycolytic metabolite redox couple lactate/pyruvate, an indirect measure for cytosolic NAD^+^/NADH ratio, was unaffected in the DRG of primed mice (Figure S2D). However, the extracellular acidification rate (ECAR), a measure of anaerobic glycolysis, was significantly increased in cultured DRG neurons isolated from primed mice compared with naive mice (Figure 2D). Overall, these data point to disturbances in glycolysis, mitochondrial respiration, nicotinamide metabolism, and mitochondrial redox balance in the DRG after hyperalgesic priming.

NAD^+^ supplementation *in vivo* can mitigate enhanced glycolysis and redox disturbances and improve mitochondrial functions.^27^^,^^28^ Moreover, systemic or oral administration of the NAD^+^ precursor NR increases NAD^+^ levels in different tissues, including nervous tissue.^29^^,^^30^ Therefore, we tested if NR supplementation is sufficient to restore resolution of PGE_2_-induced hyperalgesia in primed mice. Indeed, NR supplementation, through an intraperitoneal injection prior to intraplantar PGE_2_ injection, prevented failure to resolve PGE_2_-induced hyperalgesia in primed mice, while NR supplementation did not affect PGE_2_-induced hyperalgesia in non-primed mice (Figures 2E and S2E). Disturbed redox balance is often associated with oxidative stress, e.g., due to oversupply of NADH to the ETC, which promotes electron leakage and mitochondrial superoxide (mtROS) production.^31^ To assess mtROS production in DRG neurons, mice were injected intrathecally with MitoTrackerRedCM-H2XROS at day 7 after intraplantar carrageenan or vehicle injection.^16^^,^^32^ At day 7, when mice had recovered from inflammatory hyperalgesia, MitoTrackerRedCM-H2XROS fluorescence (Figures 2F and 2G) and the number of MitoTrackerRedCM-H2XROS-positive small-diameter DRG neurons (<20 μm) were increased (mtROS-positive/-negative neurons: non-primed 35/693 [∼5%], primed 151/1,021 [∼15%], p < 0.0001). In medium-/large-diameter neurons, MitoTrackerRedCM-H2XROS fluorescence (Figures 2F and 2G) or the number of positive neurons (>20 μm, non-primed 25/235 [∼10%], primed 82/549 [∼15%], p = 0.0912) were not significantly affected.^33^ Pharmacological blockade of superoxide, with an intrathecal injection of the mitochondrial ROS scavenger Mito-TEMPOL,^34^ prior to intraplantar injection of PGE_2_, restored resolution of PGE_2_-induced hyperalgesia in primed mice (Figures 2H and S2F). In conclusion, disturbances in redox balance and oxidative stress persist in DRG neurons after resolution of inflammatory pain. Our data suggest that these disturbances lead to failure in pain resolution after an inflammatory stimuli, driving the transition to chronic pain in primed mice.

### ATPSc-KMT promotes mitochondrial hyperactivity and induces hyperalgesic priming

**Figure 3:**
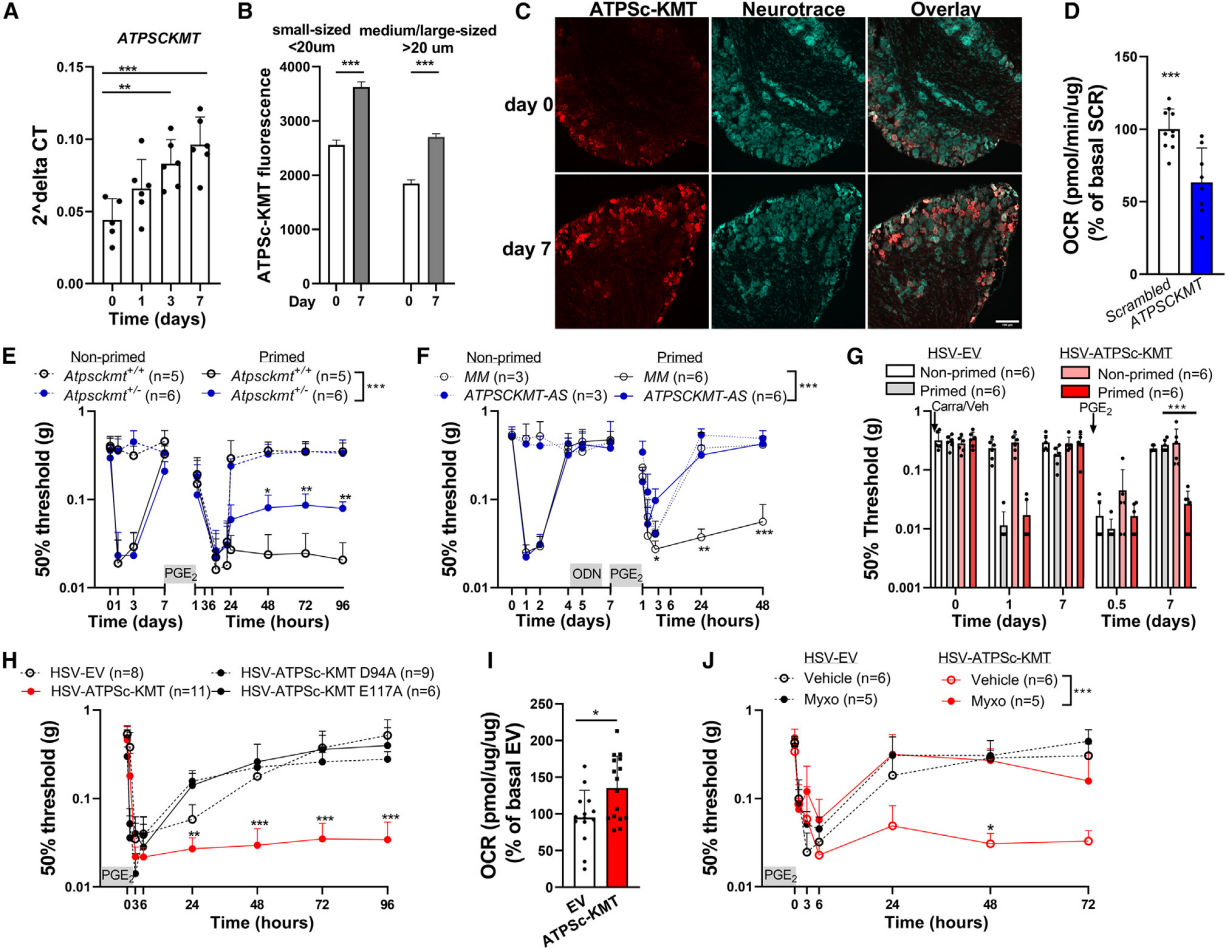
ATPSc-KMT expression promotes mitochondrial hyperactivity in DRG neurons and mimics hyperalgesic priming (A and B) Expression of ATPSc-KMT (A) mRNA in the whole DRG (n = 6) and (B) protein in soma of sensory neurons of lumbar (L3–L5) DRGs at different days after intraplantar carrageenan. Mean ATPSc-KMT fluorescence intensity in small- and medium-/large-sized neurons at indicated days (small sized n = 1,200–1,300 cells, medium/large sized = 1,230–1,325 cells). (C) Example pictures of ATPSc-KMT fluorescence in DRG neurons (scale bar: 100 μM). (D) OCR measurements in primary sensory neurons after lentiviral-mediated *ATPSCKMT* knockdown (n = 8) compared to scrambled controls (n = 10). (E–H) Course of PGE_2_-induced mechanical hyperalgesia (E) in carrageenan-primed and non-primed *Atpsckmt*^+/+^ (WT) and *Atpsckmt*^+/−^ mice, (F) after i.t. injection of *ATPSCKMT*-antisense (AS) or mismatched (MM) control oligodeoxynucleotide (ODN; 3 μg/μL, 5 μL) at days 4, 5, and 6 in carrageenan-primed and non-primed mice, and (G) after i.t. *ATPSCKMT*-AS injections (day 4, 5, and 6) in primed and non-primed mice. To reconstitute *ATPSCKMT* expression*,* mice received intraplantar injections with HSV-ATPSc-KMT (35,000 plaque-forming unit [PFU]/paw) or HSV-EV as control at days 4 and 6 after carrageenan and (H) after intraplantar HSV injections to express ATPSc-KMT or ATPSc-KMT catalytically inactive mutants (D94A or E117A) or EV in DRG neurons. Intraplantar HSV injections were administrated at days −3 and −1 (35.000 PFU/paw). (I) OCR measurements after HSV-mediated ATPSc-KMT expression (n = 16) in DRG neurons and HSV- EV expression as control (n = 14). (J) Similar as (H) but with i.t. injection of myxo (50 μM) 15 min prior to intraplantar PGE_2_. Data are represented as mean ± SD. ∗p < 0.05, ∗∗p < 0.01, ∗∗∗p < 0.001. Statistical analyses were performed by Student’s t test (D and I), one-way ANOVA (A and B) followed by Dunnett’s multiple comparison test, or two-way repeated measures ANOVA followed by a post hoc Sidak’s multiple comparison test (E–H and J; stars indicate significance comparing carrageenan- and ATPSc-KMT-primed conditions). Primed mice by ATPSc-KMT overexpression are indicated with red bars/lines, and blue bars/lines indicate *ATPSCKMT* knockdown.

ATPSc-KMT has recently been identified as a mitochondrial protein driving chronic inflammatory pain. ATPSc-KMT promotes mtROS formation when overexpressed and is required for efficient mitochondrial respiration.^16^^,^^17^ Therefore, we asked whether changes in ATPSc-KMT expression may underlie the persistent mitochondrial and metabolic adaptations that cause failure of pain resolution after priming. We first evaluated *ATPSCKMT* mRNA expression in the DRG during the course of carrageenan-induced hyperalgesia. *ATPSCKMT* mRNA expression was increased in the lumbar DRG at day 3 and 7 after intraplantar carrageenan injection (Figure 3A). At day 7, when hyperalgesia had resolved and mice were primed, the ATPSc-KMT protein level was increased both in small- and medium-/large-diameter neurons compared to vehicle-injected mice (Figures 3B and 3C). Thus, carrageenan increases ATPSc-KMT expression at mRNA and protein levels in DRG neurons, and these changes persist after inflammatory hyperalgesia has resolved.

Deficiency of *ATPSCKMT* impairs complex V activity and mitochondrial respiration in HAP1 and Neuro2a (N2A) cells.^17^ Here, we show that *ATPSCKMT* knockdown also reduced basal mitochondrial respiration, proton leakage, and ATP-driven and maximal respiration in cultured DRG neurons (Figures 3D, S3A, and S3B). To test whether *ATPSCKMT* deficiency prevents failure in resolution in PGE_2_-induced hyperalgesia in primed mice, we used global heterozygous *ATPSCKMT* knockout (*Atpsckmt*^+/−^) mice. The course of carrageen-induced hyperalgesia was similar between *Atpsckmt*^+/−^ and wild-type (WT) littermate controls (*Atpsckmt*^+/+^) mice. However, after carrageenan-induced priming, the magnitude of persistent PGE_2_-induced mechanical and thermal hyperalgesia is reduced in primed *Atpsckmt*^+/−^ mice compared with WT mice (Figures 3E and S3C). To exclude that ATPSc-KMT has major effects on electrophysical properties, we assessed small DRG neurons of *Atpsckmt*^+/−^ mice and compared them with WT littermate controls. We did not observe major differences except for a small but significant decrease in the threshold of activation in DRG neurons of *Atpsckmt*^+/−^ mice (Figure S3D). In addition, baseline intracellular Ca^2+^ levels were slightly reduced in DRG neurons of *Atpsckmt*^+/−^ mice. However, capsaicin-induced intracellular Ca^2+^ fluxes, baseline, and acute carrageenan- and PGE_2_-induced thermal and mechanical sensitivity were indistinguishable between genotypes (Figures 3E, S3E, and S3F). To further establish a role of ATPSc-KMT and investigate whether ATPSc-KMT expression in DRG sensory neurons prevents resolution of subsequent PGE_2_-induced hyperalgesia in primed mice, we targeted ATPSc-KMT expression in the lumbar DRG by using intrathecal injections of mouse *ATPSCKMT* antisense (*ATPSCKMT*-AS) oligodeoxynucleotide. To that end, mice received lumbar intrathecal injections of *ATPSCKMT*-AS at days 4, 5, and 6 after intraplantar carrageenan, a strategy that reduces *ATPSCKMT* expression and mitochondrial respiration in DRG neurons (Figure S3G).^16^ *ATPSCKMT*-AS fully restored the resolution of PGE_2_-induced hyperalgesia in carrageenan-primed mice, while PGE_2_-induced hyperalgesia did not resolve in carrageenan-primed mice treated with control mismatch oligodeoxynucleotides (MM-AS) (Figures 3F and S3H). Knockdown of *ATPSCKMT* did not affect the course of PGE_2_-induced hyperalgesia in non-primed mice (Figure 3F). To confirm neuronal specificity, we assessed whether selectively expressing human ATPSc-KMT in sensory neurons with intraplantar herpes simplex virus (HSV) amplicons^35^ would prevent mouse *ATPSCKMT-*AS-induced restoration of the resolution of PGE_2_-induced hyperalgesia in primed mice. Primed mice intrathecally injected with *ATPSCKMT*-AS subjected to intraplantar administration of HSV-ATPSc-KMT, prior to intraplantar injection of PGE_2_, continued to display PGE_2_-induced hyperalgesia, while those treated with empty vector (HSV-EV) had completely resolved from PGE_2_-induced hyperalgesia 7 days after injection (Figures 3G and S3I).

Next, we assessed whether increased ATPSc-KMT expression in DRG neurons is sufficient to mimic a hyperalgesic priming state, enhanced mitochondrial respiration, and failure in resolution of PGE_2_-induced hyperalgesia. Administration of HSV-ATPSc-KMT prior to intraplantar PGE_2_ prevented the resolution of PGE_2_-evoked mechanical (Figure 3H) and thermal hyperalgesia (Figure S3J). In contrast, resolution of PGE_2_-induced hyperalgesia was unaffected when mice were administered with HSV-EV or HSV containing two different ATPSc-KMT mutants, D94A and E117A (located in motif 1 and post I, respectively),^17^ which are catalytically inactive (Figures 3H and S3J).^16^^,^^17^ As anticipated, ATPSc-KMT overexpression was sufficient to increase basal respiration, proton leakage and ATP-driven and maximal respiration in N2A cells (Figure S3K) and cultured primary sensory neurons (Figures 3I, S3L, and S3M). The increase in mitochondrial respiration was not caused by other oxygen-consuming processes or cellular metabolism, as complex II-driven respiration in isolated mitochondria was increased by ATPSc-KMT overexpression (Figure S3N). In addition, injection of the complex III inhibitor myxothiazol restored the resolution of PGE_2_-induced hyperalgesia in mice overexpressing ATPSc-KMT (Figure 3J). These data indicate that elevated ATPSc-KMT expression in DRG neurons is sufficient to mimic the primed states of sensory neurons after a transient inflammatory stimulus by promoting mitochondrial hyperactivity and causing failure in the resolution of PGE_2_-induced hyperalgesia.

Next, we aimed to understand how ATPSc-KMT expression increases mitochondrial activity and impairs resolution of PGE_2_-induced inflammatory hyperalgesia. Recently, we showed that ATPSc-KMT methylates Lys-43 of ATPSc (complex V of the ETC), suggesting that alterations in the methylation status of Lys-43 may regulate pain resolution.^17^ However, we found that Lys-43 in ATPSc was already fully methylated (trimethylated) in the DRG from naive mice (Figures S3O and S3P), making it unlikely that increased ATPSc-KMT activity/expression enhances mitochondrial respiratory activity through increased methylation of Lys-43. In addition, we observed that when ATP synthase was fully inhibited and uncoupled from OXPHOS, *ATPSCKMT*-deficient (ATPSc-KMT^−/−^) HAP1 cells had significantly reduced OCRs compared with WT cells and also reduced OCRs compared with ATPSc-KMT^−/−^ cells reconstituted with ATPSc-KMT WT but not those reconstituted with catalytically inactive ATPSc-KMT-E117A mutant (Figure S3Q). These data suggest that ATPSc-KMT may regulate mitochondrial activity by mechanism(s) other than methylation of ATPSc.

### ATPSc-KMT expression affects cellular metabolism and disturbs redox balance

**Figure 4:**
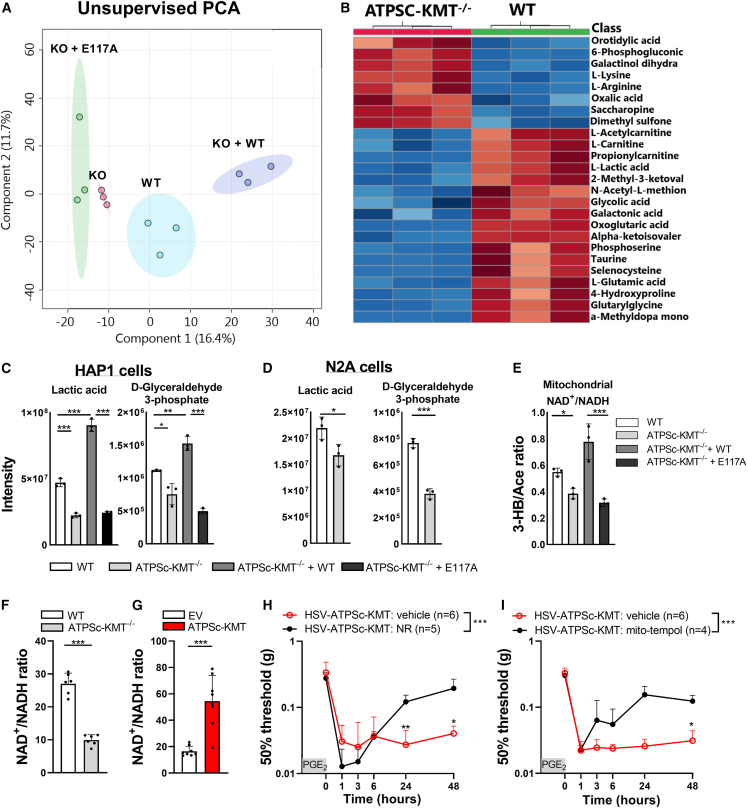
ATPSc-KMT affects cellular metabolism and redox balance (A) Unsupervised principal-component analysis (PCA) of whole metabolomics from DI-HMRS dataset of HAP1 cells expressing ATPSc-KMT (WT), cells deficient of ATPSc-KMT (knockout [KO]), and KO cells reconstituted with WT (KO + WT) or with catalytically inactive ATPSc-KMT mutant (KO + E117A). (B) Heatmap of metabolite levels that were significantly changed between WT and ATPSc-KMT^−/−^ cells. Blue represents reduced intensity, and red represents increased intensity. (C and D) Intensity of lactic acid and GA3P in (C) HAP1 cells and (D) N2A cells that are ATPSc-KMT proficient or deficient. (E) Direct ratio of 3-hydroxybutyrate to acetoacetate measured with DI-HMRS as proxy for mitochondrial NAD^+^/NADH ratio. (F and G) NAD^+^/NADH ratio calculated from direct measurement of cellular NAD^+^ and NADH in (F) HAP1 WT and ATPSc-KMT-deficient cells and (G) in N2A cells after overexpression of ATPSc-KMT or control EV. (H) Course of PGE_2_-induced mechanical hyperalgesia after an intraperitoneal injection of NR, 15 min prior to intraplantar PGE_2_ injection, in mice overexpressing ATPSc-KMT in DRG neurons. Intraplantar HSV injections were administrated at days −3 and −1 (35.000 PFU/paw). (I) Same as in (H) but after i.t. injection of Mito-TEMPOL, 15 min prior to intraplantar PGE_2_ injection. Data are represented as mean ± SD. ∗p < 0.05, ∗∗p < 0.01, ∗∗∗p < 0.001. Statistical analyses were performed by Student’s t test (D, F, and G), one-way ANOVA (C and E), or two-way repeated measures ANOVA followed by a post hoc Sidak’s multiple comparison test (H and I).

To assess whether ATPSc-KMT expression affects cellular metabolism, we performed non-quantitative DI-HRMS. An unsupervised principal-component analysis (PCA) showed that ATPSc-KMT^−/−^ HAP1 cells clustered distinctly from WT cells and that ATPSc-KMT^−/−^ cells reconstituted with WT ATPSc-KMT. ATPSc-KMT^−/−^ cells reconstituted with the catalytically inactive mutant E117A clustered close to ATPSc-KMT-deficient cells (Figure 4A). Enrichment analysis identified a group of functionally related metabolites and ranked metabolites involved in the mitochondrial ETC and the Warburg effect (high rate of glycolysis) at the top of the list (Figure S4A). Heatmap analysis indicates that L-acetylcarnitine, which buffers the pool of acetyl-CoA to enter the Krebs (TCA) cycle,^36^ was significantly reduced in ATPSc-KMT-deficient cells and normalized again by reconstitution with WT ATPSc-KMT (Figures 4B and S4B). Similarly, D-glyceraldehyde 3-phosphate (GA3P) and lactic acid, the intermediate and end-product metabolites of anaerobic glycolysis, respectively, were decreased 2-fold in ATPSc-KMT^−/−^ cells compared with WT cells and recovered by reconstitution with WT ATPSc-KMT but not with ATPSc-KMT-E117A mutant (Figure 4C). The decrease of lactate was validated in a targeted quantitative analysis of TCA metabolites (Figure S4C) and in CRISPR-Cas9-generated ATPSc-KMT-deficient N2A cells (Figure 4D). Decreased lactate levels are indicative of impaired glycolysis.^26^ In accordance, ECAR levels, as a proxy of glycolysis, were reduced in ATPSc-KMT^−/−^ cells compared with WT cells but were restored after reconstitution with WT, but not E117A-mutated ATPSc-KMT (Figure S4D), indicating that ATPSc-KMT influences glycolysis. These data confirm our hypothesis that ATPSc-KMT expression changes cellular metabolism in HAP1 and N2A cells by promoting glycolysis and OXPHOS.

Since NR in DRG neurons was reduced after resolution of carrageenan-induced hyperalgesia at a time point when ATPSc-KMT expression was increased, we investigated whether NAD^+^ precursors are affected by ATPSc-KMT. ATPSc-KMT expression and activity did not affect NR, quinolinic acid, or nicotinic acid levels (Figure S4E). However, ATPSc-KMT deficiency decreased the 3-hydroxybutyrate/acetoacetate ratio, which was restored with WT ATPSc-KMT but not with the ATPSc-KMT-E117A mutant (Figure 4E). These data suggest that ATPSc-KMT may disturb the mitochondrial NAD^+^/NADH ratio. Decreased NADH levels are not favorable in mitochondria since NADH is an important complex I substrate for the ETC to produce ATP. In line with the indirect measurements that pointed toward a shift in the mitochondrial NAD^+^/NADH ratio (Figure 4E), direct NAD^+^ and NADH measurements with ultra-high-performance liquid chromatography (UPLC) showed that the total cellular NAD^+^/NADH ratio was reduced in ATPSc-KMT-deficient HAP1 cells (Figure 4F). Conversely, transient overexpression of ATPSc-KMT in N2A cells increased the cellular NAD^+^/NADH ratio (Figure 4G) by predominately decreasing the NADH cellular pool (Figure S4F). Likely, this shift is caused by the increased consumption of NADH due to the ATPSc-KMT-induced increase in OXPHOS (Figures 3I and S3K–S3N). Next, we investigated whether NR supplementation was also sufficient to restore resolution of PGE_2_-induced hyperalgesia in HSV-ATPSc-KMT-primed mice. An intraperitoneal injection of NR prior to intraplantar PGE_2_ injection completely prevented the failure in resolving the PGE_2_-induced mechanical hyperalgesia in HSV-ATPSc-KMT-primed mice (Figures 4H and S5A).

Redox balance controls NADPH-requiring antioxidant pathways.^37^ Therefore, a redox imbalance (e.g., an increased mitochondrial NAD^+^/NADH ratio) impairs the cellular antioxidant capacity, leading to oxidative stress.^28^^,^^38^ Accordingly, ATPSc-KMT-deficient cells, which had a reduced NAD^+^/NADH ratio (Figures 4E and 4F), also had decreased mtROS levels compared with WT cells or ATPSc-KMT^−/−^ cells reconstituted with WT ATPSc-KMT but not with the catalytically inactive ATPSc-KMT-E117A mutant (Figure S5B). Moreover, intrathecal injection of the mitochondrial ROS scavenger Mito-TEMPOL, prior to intraplantar PGE_2_ injection in mice expressing HSV-ATPSc-KMT, restored the resolution of PGE_2_-induced hyperalgesia (Figures 4I and S5C). Overall, these data suggest that increased ATPSc-KMT MTase activity affects the formation of metabolites, increases oxidative stress, and disturbs the redox state of sensory neurons. As a consequence, resolution of PGE_2_-induced hyperalgesia fails. However, NAD^+^ supplementation or blocking the oxidative stress helps in restoring the resolution of PGE_2_-induced hyperalgesia in mice expressing HSV-ATPSc-KMT.

### NAD^+^ supplementation attenuates chronic inflammatory pain

**Figure 5:**
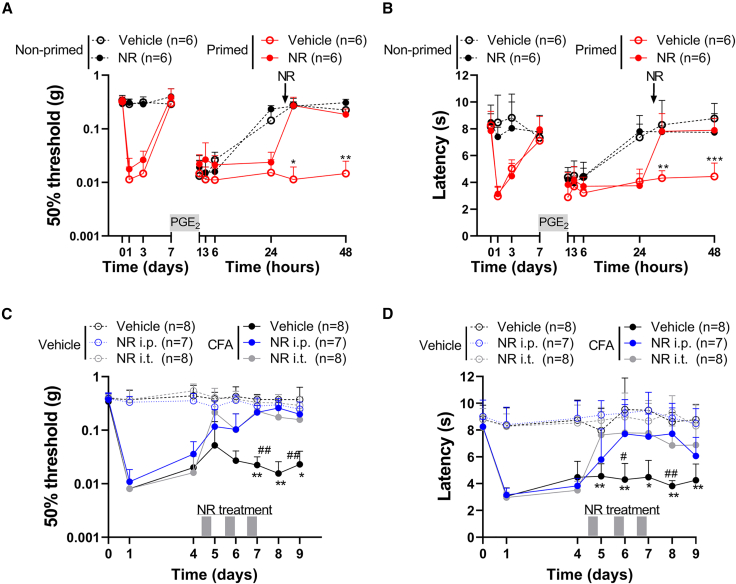
NAD^+^ supplementation attenuates primed and CFA-induced persistent pain (A and B) Mice received intraperitoneal (i.p.; 500 mg/kg) injection with NR 24 h after intraplanar PGE_2_. (C and D) Mice received i.p. (500 mg/kg) or i.t. (50 μg) injections with NR at days 5, 6, and 7 after intraplantar complete Freund’s adjuvant (CFA) or vehicle (∗i.t. vehicle versus NR, #i.p. vehicle versus NR). (A and C) Mechanical and (B and D) thermal hyperalgesia was measured 4 h after each NR administration. Data are represented as mean ± SD. ∗p < 0.05, ∗∗p < 0.01, ∗∗∗p < 0.001. Statistical analyses were performed by two-way repeated measures ANOVA followed by a post hoc Sidak’s multiple comparison test.

Since our data indicate that NR supplementation is sufficient to restore resolution of PGE_2_-induced hyperalgesia in carrageenan-primed mice, or mice overexpressing ATPSc-KMT, we next tested whether NR supplementation would be sufficient to resolve prolonged PGE_2_-induced hyperalgesia in primed mice. One intraperitoneal injection of NR at 24 h after intraplantar PGE_2_ injection attenuated mechanical and thermal hyperalgesia in primed mice. NR supplementation did not affect PGE_2_-induced hyperalgesia in non-primed mice (Figures 5A and 5B). Given that nicotinamide not only has effects on metabolism but also on neuronal development and survival,^39^ we wanted to verify neuronal integrity to address whether the non-resolving pain hypersensitivity could be caused by neuronal damage. To that end, we measured ATF3, a marker of neuronal damage. Importantly, we did not observe signs of nuclear ATF3 in DRG neurons after priming (Figure S5D). These findings are in line with earlier findings that the primed state can be masked or unmasked by using a second stimulus,^19^^,^^40^ suggesting that the non-resolving mechanical hypersensitivity is likely not the result of damaged neurons.

Next, we tested efficacy of NR in another chronic inflammatory pain model, where persistent inflammatory hyperalgesia was induced by an intraplantar injection of complete Freund’s adjuvant (CFA).^16^ 5 days after development of CFA-induced inflammatory pain, mice received intraperitoneal (i.p.) injections with NR for 3 consecutive days. NR administration attenuated CFA-induced mechanical (Figure 5C) and thermal (Figure 5D) hyperalgesia compared to vehicle-treated mice, whereas NR treatment did not have any effect on pain associated behaviors in mice that received an intraplantar vehicle injection only. Local intrathecal (i.t.) injection with NR, to target the DRG and spinal cord but not the inflamed tissue, also resolved CFA-induced persistent inflammatory pain (Figures 5C and 5D), indicating that the inhibition of CFA-induced inflammatory pain is likely not due to NR targeting CFA-induced inflammation in the hind paw.

## Discussion

**Figure 6:**
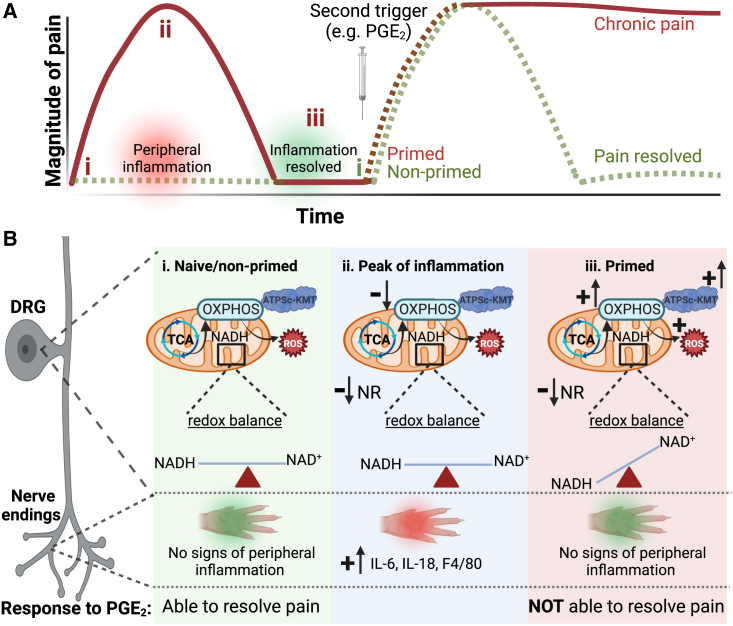
Schematic overview of proposed mechanisms (A) Time course of mechanical hypersensitivity in (i) naive mice and after intraplantar carrageenan, at the peak of (ii) acute pain and (iii) when hyperalgesia has resolved (primed state). A second stimulus (PGE_2_) is given to primed and non-primed mice, where inflammatory hyperalgesia fails to resolve when mice were primed. (B) A summary of our main findings at the level of DRG and paw at the different stages. Compared to naive mice and or non/primed mice (i), at the peak of transient inflammatory pain (ii), there is a clear increase in inflammatory markers in the inflamed paw and a reduction in OXPHOS without disturbance in redox balance in the DRG. (iii) When carrageenan-induced inflammatory hyperalgesia has resolved, signs of paw inflammation have disappeared. At this stage, ATPSc-KMT expression and OXPHOS are increased in DRG neurons, concurrent with signs of oxidative stress (ROS production), reduced NR levels, and an increased mitochondrial NAD^+^/NADH ratio, possibly as a consequence of increased NADH consumption by enhanced activity of the ETC. These peripheral inflammation-induced mitochondrial and metabolic disturbances in the DRG lead to the inability to resolve from a subsequent inflammatory stimulus, like PGE_2_. Arrows with a plus (+) symbol indicate increase/upregulation, while the arrows with a minus (−) symbol suggest reduction in expression/activity. Figure was created with BioRender.com.

The mechanisms that impair resolution of inflammatory pain leading to persisting pain are still poorly understood. We observed that after resolution of inflammatory hyperalgesia, when latent plasticity of the sensory system is present, mitochondrial and metabolic changes persist in the DRG. These changes pointed to a disturbed redox balance. Importantly, these mitochondrial and metabolic disturbances are fundamental to the inability of primed mice to resolve from PGE_2_-induced inflammatory hyperalgesia (Figure 6). Notably, mitochondrial hyperactivity, metabolic disturbances, and priming-induced failure in resolution of PGE_2_-induced hyperalgesia were fully mimicked by increasing the expression of *ATPSCKMT* in DRG neurons. Attenuating mitochondrial hyperactivity, scavenging mtROS, or NAD^+^ supplementation was sufficient to restore failed resolution of PGE_2_-induced hyperalgesia, and NAD^+^ supplementation even promoted pain resolution in a model of persistent inflammatory pain. Overall, these results highlight the importance of tight control of mitochondrial and metabolic activity in DRG neurons to ensure resolution of inflammatory pain.

Priming induced by peripheral transient inflammation causes neuroplasticity of nociceptors, which includes increased mRNA translation and switch in cAMP signaling toward a PKCε-dependent pathway.^18^^,^^41^ The involvement of mitochondria in hyperalgesic priming has been previously postulated, as several mitochondrial proteins are targets of PKCε signaling.^42^ However, earlier studies have excluded a potential role of mitochondria in peripheral nociceptor endings to restore failure in PGE_2_-induced hyperalgesia.^43^^,^^44^ Nonetheless, mitochondria in the soma of nociceptors during hyperalgesic priming were not investigated. We hypothesized that the excitable soma of sensory neurons^45^ is the ideal place to integrate signals and process axonal activity to initiate (transcriptional) adaptations to support altered neuronal functioning. Given this central role of the soma and the fact that mitochondria are mainly formed at the soma,^46^ mitochondrial fitness in the soma is key to maintain bioenergetics and cellular homeostasis and to ensure the ability to adequately respond to noxious triggers. Indeed, we found that peripheral inflammation reduced mitochondrial respiration in both the soma of DRG neurons and sciatic nerves. However, after resolution of hyperalgesia and inflammation, mitochondrial respiration was increased in the soma but not in the axons. Moreover, inhibition of mitochondrial activity in the DRG, but not in the hind paw with nerve endings, restored resolution of inflammatory pain. Thus, these results support the hypothesis that mitochondria are distinctly regulated in the soma of DRG neurons compared to sciatic nerves and that their peripheral endings and mitochondria in the soma play a unique role in hyperalgesic priming.^47^

We observed that DRG neurons maintain a higher mitochondrial respiration after resolution of a peripheral inflammation. Why would neurons benefit from doing so? Various cell types, such as stress-induced murine and human fibroblasts, DRG neurons, and peripheral blood mononuclear cells of patients with diabetes mellitus or rheumatoid arthritis with an active disease increase their OXPHOS, possibly to compensate for a transient decline in NAD^+^ or ATP, to prevent oxidative stress and to promote cellular homeostasis.^48^^,^^49^^,^^50^ The observed persistent increase of OXPHOS in DRG neurons of primed mice possibly promotes the generation of ATP to fuel energy-consuming processes, such as the transport of proteins/organelles along the cytoskeleton or to support increased protein translation, processes all needed to maintain the primed state.^51^^,^^52^ Therefore, we hypothesize that the inflammation-induced persisting increase in sensory neuron OXPHOS is likely needed to maintain the newly introduced homeostatic set point of primed neurons to permit this kind of “neuronal memory” to respond differently to a future stimulus.

The precise mechanisms that maintain the enhanced OXPHOS in sensory neurons after priming are not fully understood. However, we found that carrageenan induced an increase in ATPsc-KMT expression in sensory neurons, which may be sufficient to increase OXPHOS.^17^ Similarly, ATPSc-KMT mRNA expression in the DRG is also increased in a model of CFA-induced persistent inflammatory pain, whereas the pain is attenuated by either inhibition of complex I of the ETC or *ATPSCKMT* knockdown.^14^^,^^16^ These findings suggest that upregulation of ATPSc-KMT expression occurs more broadly and is linked to changes in mitochondrial respiration, possibly to support cellular energy requirements after an inflammatory trigger.

The question arises how ATPsc-KMT promotes OXPHOS activity. ATPsc-KMT methylates Lys-43 of ATPSc (part of complex V of the ETC), thereby regulating OXPHOS activity in several cell lines and primary neurons.^17^ However, Lys-43 is already fully methylated in the DRG of naive mice, making it unlikely that ATPsc-KMT enhanced the OXPHOS via Lys-43 methylation in DRG neurons of primed mice. Possibly, ATPsc-KMT enhances OXPHOS by interacting with and possibly methylating other substrates. Indeed, when ATP synthase was fully inhibited and uncoupled from OXPHOS, *ATPSCKMT*-deficient (ATPSc-KMT^−/−^) HAP1 cells still had a significantly reduced OCR compared to WT cells. This suggests that other ATPSc-KMT interactants/substrates are possibly involved in regulating OXPHOS, which remains to be elucidated.

Alterations in OXPHOS often generate ROS as a byproduct, which may result in oxidative stress and a disturbed redox balance.^48^^,^^53^ We show here that disturbed mitochondrial NAD^+^/NADH redox balance persists in the DRG beyond resolution of inflammation. But how are changes in the redox state associated with pain? It is well known that a drop in cellular NAD^+^ level has detrimental effects for many biological processes. For example, a decline in NAD^+^ is associated with several pathologies, including neurodegenerative diseases.^24^ In models of nerve injury and chemotherapy-induced chronic pain, NAD^+^ levels are reduced in sciatic nerves and DRG neurons.^54^^,^^55^^,^^56^^,^^57^ In DRG lysates of primed mice, the NAD^+^ precursor NR was reduced, and the mitochondrial redox balance was disturbed. These changes occurred in parallel with increased mtROS levels in sensory neurons of primed mice. Thus, possibly, ROS production drives the excitability of sensory neurons. Indeed, redox alterations induce long-term potentiation in neurons and/or sensitize neurons through, e.g., transient receptor potential (TRPA1) and calcium channels (Cav3.2), which are involved in mechanical and cold hypersensitivity.^58^^,^^59^^,^^60^^,^^61^ Changes in cellular NAD^+^ concentrations can also directly affect neuronal excitability. As an example, reduced cellular NAD^+^ levels inactivate sodium-activated potassium channels in DRG neurons (e.g., the Slack K_NA_ channel), which leads to enhanced responsiveness of nociceptors to noxious stimuli.^62^^,^^63^ Possibly, NR supplementation dampens persistent neuronal activity after priming through the regulation of sodium-activated potassium channels. Overall, future work is needed to study the interplay of NR and different ion channel activities to understand their link with DRG neuronal excitability and chronic pain.

The upstream trigger leading to changes in mitochondrial function and metabolism in DRG neurons is still unclear. Infiltration of immune cells, such as macrophages, are observed in paws and the DRG from day 1 until day 3 after intraplanar carrageenan.^7^ Macrophages can release a variety of cytokines, including but not limited to interleukin-33 (IL-33), IL-1β, and interferon γ (IFNγ). Several of these cytokines, such as IL-33 and IFNγ, are known to affect mitochondrial metabolism in immune cells.^64^^,^^65^ Moreover, various cytokines may directly affect mitochondrial respiration and mtROS and inflammasome activation in neurons (Saleh et al.^66^ and Silva et al.^67^). Of note, i.t. IL-33 promotes priming and prolongs PGE_2_-induced hyperalgesia, compared to non-primed mice, by binding to its receptor ST2,^68^ suggesting that IL-33 may be a critical upstream cytokine that could induce mitochondrial changes in sensory neurons. Future work is needed to identify critical upstream events that lead to these changes in mitochondrial functions in neurons.

A limitation of this study is that we used AS oligodeoxynucleotide (ODN)-mediated *ATPSCKMT* knockdown and i.t. administration routes to target DRG neurons. These methods are not specific to neurons only and could potentially also affect other cells in the DRG or even in the spinal cord. However, i.t. AS ODN administrations significantly reduced *ATPSCKMT* expression in the DRG and not in the spinal cord.^16^ Additionally, we selectively rescued ATPSc-KMT expression in sensory neurons in *ATPScKMT*^+/−^ mice by intraplantar injection of HSV-ATPsc-KMT, which selectively targets the sensory neurons. Our earlier work showed that HSV-ATPSc-KMT does not cause detectable expression in cells other than sensory neurons.^16^ Along these lines, we measured the metabolic profile in total DRG lysates, as this is technically difficult to measure specifically in isolated DRG neurons. Thus, we cannot fully exclude the possibility that observed metabolic differences occurred in cells other than DRG neurons, such as glia cells or immune cells. Importantly, the numbers of immune cells in the DRG of primed mice (day 7 after carrageenan) did not differ from that in the DRG of naive mice,^7^ which supports that the changes in metabolites and redox balance are likely not caused by changes in the number of immune cells in the DRG but rather by metabolic changes within DRG neurons. Although we cannot fully exclude that metabolic alterations occurred within other cells in the DRG, sensory neurons likely contribute the most to these changes, and they constitute the majority of cells in the DRG.^69^ Moreover, ATPSc-KMT expression, glycolysis, and mtROS formation were increased specifically in DRG neurons, suggesting that sensory neurons are the main contributors for the observed mitochondrial and metabolic changes within the DRG.

Various studies describe that NAD^+^ precursors, including NR, protect neurons when they are injured (e.g., Parkinson, Alzheimer’s disease).^70^ However, whether NAD^+^ or its precursors have analgesic potential is less clear. Here, we observed that supplementation of the NAD^+^ precursor NR was sufficient to restore pain resolution not only in primed mice but also in CFA-induced persistent inflammatory pain, a model known to be associated with mitochondrial abnormalities in the DRG.^14^ Oral administration of NR also reverses chemotherapy-induced and diabetic-induced neuropathy in rodents.^55^^,^^71^ There are several mechanisms that could explain how NAD^+^ promotes mitochondrial fitness and cellular homeostasis. The most described mechanism of action of NAD^+^ supplementation is the regulation of redox balance and antioxidant activity.^23^^,^^27^^,^^72^ Another proposed mechanism is that NR improves mitochondrial fitness by activating cellular metabolic NAD^+^ sensors, such as sirtuins (NAD^+^-dependent histone deacetylase), which promote expression of antioxidant genes and genes involved in mitochondrial biogenesis.^73^^,^^74^ Moreover, NR was reported to promote mitophagy, i.e., removal of damaged mitochondria, in a neurodegenerative mouse model.^30^ Thus, NR supplementation possibly facilitates mitochondrial quality control mechanisms in neurons that may overcome the observed priming-induced mitochondrial alterations. Overall, supplementation with NAD^+^ precursors, including NR, may have clinical benefit to treat chronic pain. Recent pharmacokinetic studies already proved that NR is safe and well tolerated in humans.^72^^,^^75^ A small pilot study from 1959 described that NAD^+^ therapy induced remission and reduced pain outcomes in patients with arthritis.^76^ However, a follow-up pilot study in 1996 reported that nicotinamide treatment did not change pain levels but did improve joint flexibility and reduced inflammation, allowing patients with osteoarthritis to reduce their anti-inflammatory medication.^77^ Larger clinical trials are needed to investigate whether NAD^+^ supplementation may hold promise for treatment of chronic pain in inflammatory diseases.

In conclusion, here, we provide evidence that peripheral inflammation induces persistent mitochondrial and metabolic changes in the soma of sensory neurons, which affected the ability to resolve from hyperalgesia induced by a subsequent inflammatory trigger. Thus, metabolic changes in sensory neurons result in failure of endogenous pain resolution pathways and drive the transition to chronic pain. Importantly, targeting mitochondrial respiration, scavenging ROS, or supplementing NR represent potential therapeutic strategies to restore failing pain resolution pathways to treat chronic inflammatory pain.

### Limitations of the study

Although we did not observe that a reduction in ATPSc-KMT expression in DRG neurons affected electrophysiological properties, we did not evaluate whether overexpression of ATPSc-KMT would affect electrophysiological functions of sensory neurons and potentially impact resolution of PGE_2_-induced inflammatory hyperalgesia. In addition, ATPSc-KMT could potentially affect downstream signaling from PGE_2_, thereby causing failing pain resolution, as earlier research has shown that biased PGE_2_ signaling induces prolonged mechanical hypersensitivity.^18^^,^^78^ Our work clearly demonstrated that an increase in ATPSc-KMT expression was sufficient to affect the metabolic state of sensory neurons and to promote failure in pain resolution. This work did not reveal whether these levels of ATPSc-KMT and associated perturbations in metabolism need to persist to prevent resolution of PGE_2_-induced hyperalgesia or whether a non-persisting increase in these parameters is sufficient to predispose to a failure in pain resolution.

## STAR★Methods

### Key resources table

**Table:** 

REAGENT or RESOURCE	SOURCE	IDENTIFIER
**Antibodies**		

CD45 (APC-eF780; clone: 30-F11)	eBioscience	47-0451-82, RRID:AB_1548781
ef506 viability marker	eBioscience	15560607
Anti-OxPhos Rodent WB Antibody Cocktail	Thermo Fisher Scientific	45–8099; RRID:AB_458099
Anti- beta-Actin antibody	Abcam	ab8229; RRID: AB8229
Anti-ATPSCKMT	Biorbyt	orb183565; RRID:AB_orb183565
Anti-Goat (AF488; clone: Donkey)	LifeTech	A11055; RRID: AB_A11055
Anti-Rabbit (AF594; clone: Donkey)	LifeTech	A21207; RRID: AB_A21207
Anti-Rabbit (AF568; clone: Donkey)	LifeTech	A10042; RRID: AB_A10042
Anti-Rat (AF647; clone: Goat)	LifeTech	A21247; RID: AB_A21247
Anti-ATF3	Thermo Fisher Scientific	PA5-101089; RRID:AB_PA5-101089

**Bacterial and virus strains**		

Recombinant HSV	In this study	N/A

**Chemicals, peptides, and recombinant proteins**		

Carrageenan	Sigma-Aldrich	22049-5G
Complete Freud’s Adjuvant	Sigma-Aldrich	F5881-10ML
PGE_2_	Merck	P0409-1mg
MitoTracker™ Red CM-H2Xros	Thermo Fisher Scientific	M7513
Myxothiazol	Sigma-Aldrich	T5580
Oligomycin	Cayman Chemicals	A579-13-5
FCCP	Sigma-Aldrich	C2920-10MG
Rotenone	Sigma-Aldrich	R8875-1G
Antimycin A	Sigma-Aldrich	A8674-25MG
NeuroTrace™ 435/455 Blue	Thermo Fisher Scientific	N21479
NeuroTrace™ 640/660 Deep-Red	Thermo Fisher Scientific	N21483
Triton X-100	Sigma-Aldrich	X100-500ML
Tween 20	Sigma-Aldrich	P1379-L
Digitonin	Sigma-Aldrich	D141
*n*-Dodecyl-β-D-Maltoside	Thermo Fisher Scientific	89902
Protease Inhibitor Cocktail	Sigma-Aldrich	P8340
Chymotrypsin Sequencing Grade	Sigma-Aldrich	11418467001
IMDM, GlutaMAX™ Supplement	Thermo Fisher Scientific	31980022
Fetal Bovine Serum, qualified, heat inactivated	Thermo Fisher Scientific	10500064
Penicillin-Streptomycin (10,000 U/mL)	Thermo Fisher Scientific	15140122
Normal donkey serum	Jackson Immunoresearch	017-000-121
β-Nicotinamide adenine dinucleotide sodium salt	Sigma-Aldrich	N0632-1G
NADH, Grade II, disodium salt	Roche Diagnostics GmbH	10128023001
b-Nicotinamide Adenine Dinucleotide-d4 (major)	Toronto Research Chemicals	N407783
Formic acid 99% ULC/MS - CC/SFC	Biosolve BV	06914143
Acetonitrile ULC/MS - CC/SFC	Biosolve BV	01204102
Methanol absolute ULC/MS - CC/SFC	Biosolve BV	13684102
Labeled amino acids standards set A	Cambridge Isotope Laboratories, Inc.	NSK-A
Labeled carnitine standards set B	Cambridge Isotope Laboratories, Inc.	NSK-B
Glycine	Merck	100590
Capsaicin	Sigma-Aldrich	M2028
Fura-2, AM	Thermo Fisher Scientific	F1221
NaCL	Sigma-Aldrich	7647-14-5
KCL	Merck	4936
HEPES	Thermo Fisher Scientific, Gibco	15630–049
D-(+)-Glucose	Sigma-Aldrich	G8270
NaOH	Merck	1310-73-2
Collagenase type XI	Sigma-Aldrich	C7657
Dispase	Thermo Fisher Scientific, Gibco	17105–041
Poly-L-Lysine	Sigma-Aldrich	P9155
Laminin	Sigma-Aldrich	L2020

**Critical commercial assays**		

SYBR Select Master Mix	Applied biosystems	4472908
Seahorse Xfe24 FluxPak	Agilent Technologies	102340–100

**Experimental models: Cell lines**		

Neuro 2A	ATCC	ATCC-CLL-131
HAP1 WT (wild-type parental cell line)	Horizon Discovery	C631
HAP1 *ATPSCKMT/FAM173B* KO cells	Horizon Discovery	HZGHC000533c006
HAP1 *ATPSCKMT/FAM173B* KO cells complemented with human *ATPSCKMT-3xFLAG*, either WT or E117A-mutated	Malecki et al.^17^	N/A

**Deposited data**		

Metabolomics data (related to Figures 2 and 4)	Dataverse.nl	https://doi.org/10.34894/MRMGNW

**Experimental models: Organisms/strains**		

Mouse: C57Bl/6JRj	Janvier	https://janvier-labs.com/en/fiche_produit/2_c57bl-6jrj_mouse/
Mouse: C57BL/6NJ-Atpsckmtem1(IMPC)J/Mmjax	Jackson Laboratory	051063-JAX

**Oligonucleotides**		

For quantitative polymerase chain reaction primers, see text	This paper	N/A
For phosphorothioated antisense oligonucleotides, see text	This paper	N/A

**Recombinant DNA**		

HSV plasmid S0109-EV	Roy et al.^79^	N/A
HSV plasmid S0109- ATPSc-KMT	Willemen et al.^16^	N/A

**Software and algorithms**		

ImageJ	N/A	N/A
MetaboAnalyst	https://www.metaboanalyst.ca	N/A
Diva	BDbiosciences	N/A
Graphpad Prism 8.3	Graphpad	N/A
Qual Browser	Thermo Fisher Scientific	v2.0.7
Proteome Discoverer	Thermo Fisher Scientific	N/A
Exactive Tune Software (version 2.9.0)	Thermo Fisher Scientific	N/A
Chipsoft (version 8.3.1)	Advion Biosciences	N/A
Xcalibur software (version 3.0)	Thermo Fisher Scientific	N/A
Thermo TraceFinder™ 4.1	Thermo Fisher Scientific	N/A
HEKA Patchmaster 2x90.2	Multichannel Systems MCS GmbH	N/A

**Other**		

LSRFortessa flow cytometer	BDbiosciences	https://www.bdbiosciences.com/en-nl/products/instruments/flow-cytometers/research-cell-analyzers/bd-lsrfortessa
Soniprep 150	Imgen Technologies	https://bioequipment-scientific.com/MSE-Soniprep-150-Plus-Ultrasonic-Disintegrator
Von Frey	Stoelting, Wood Dale, IL, US	https://stoeltingco.com/Neuroscience/Touch-Test-Sensory-Probes∼9834
Hargreaves	IITC Life Science	https://www.iitcinc.com/Product%20pages/Analgesia/Plantar.html
Zeiss Axio Observer	Zeiss, Oberkochen, Germany	N/A
QuantStudio 12K Flex	AB Instruments	N/A
StepOnePlus Real-time PCR system	AB Instruments	N/A
Seahorse Bioscience XFe24 Analyzer	Agilent Technologies	https://www.agilent.com/en/product/cell-analysis/real-time-cell-metabolic-analysis/xf-analyzers
Sunshell RP-Aqua column(150 mm × 3 mm i.d., 2.6 μm)	ChromaNik Technologies Inc.	N/A
Ultimate 3000 UHPLC system	Thermo Fisher Scientific	N/A
Q Exactive™ HF hybrid quadrupole-Orbitrap mass spectrometer	Thermo Fisher Scientific	N/A
TriVersa NanoMate	Advion Biosciences	N/A
ESI Chip	Advion Biosciences	1003446
AcroPrep 96 Filter Plates, 1 mL - 0.2 μm, PTFE membrane (5/pkg)	Pall Corporation	5055
Armadillo PCR Plate, 96-well, clear, clear wells	Thermo Fisher Scientific	AB2396
HEKA EPC 10 USB Single patch clamp amplifier	Multichannel Systems MCS GmbH	N/A

### Resource availability

#### Lead contact

Further information and requests for resources and reagents should be directed to the lead contact, Hanneke L.D.M. Willemen (h.l.d.m.willemen@umcutrecht.nl).

#### Materials availability

This study did not generate any new unique reagents.

#### Data and code availability

The DI-HMRS produced metabolomics data have been deposited in our institution preferred repository (dataverse) with identifier https://doi.org/10.34894/MRMGNW. Any additional information required to reanalyze the data reported in this work paper is available from the lead contact upon request.

### Experimental models and study participant details

#### Animals

Experiments were conducted using adult male and female (aged 8–16 weeks) C57BL/6 mice (Janvier laboratories) or C57BL/6NJ-Atpsckmt^em1(IMPC)^J/Mmjax mice (Jackson laboratories). Mice were maintained in the animal facility of the University of Utrecht and housed in groups under a 12h:12h light:dark cycle, with food and water available *ad libitum*. The cages contained environmental enrichment, including tissue papers and shelter. All experiments were performed in accordance with international guidelines and approved by the experimental animal committee of University Medical Center Utrecht (2014.I.08.059) or by the local experimental animal welfare body and the national Central Authority for Scientific Procedures on Animals (CCD, AVD115002015323 & AVD11500202010805).

Mice received an intraplantar injection (unilateral or in both hind paws) of 5 μL λ−carrageenan (primed, 1% w/v, Sigma-Aldrich) to induce transient inflammatory hyperalgesia. Non-primed mice received an intraplantar injection with saline. Day 7 after carrageenan or saline, mice were injected intraplantar with PGE_2_ (100 ng/paw, Sigma-Aldrich). For the induction of chronic inflammatory pain, mice received a unilateral intraplantar injection with 20 μL Complete Freund’s Adjuvant ((CFA), Sigma-Aldrich). Heat withdrawal latency times were determined using the Hargreaves test (IITC Life Science).^80^ Mechanical thresholds were determined using the von Frey test (Stoelting) with the up-and-down method previously described.^81^ In experiments in which mice received only intraplantar injections, each paw was considered an independent measurement in terms of latency times and 50% thresholds. In experiments with intrathecal or intraperitoneal drug administration, each animal was considered as independent measurement, so the average of the left and right paw were used. To minimize bias, animals were randomly assigned to the different groups prior to the start of experiment, and all experiments were performed by operators blinded to the treatments and/or genotypes.

#### Cell lines and primary cell cultures

Mouse neuroblastoma Neuro2a (N2A) and HEK293 cells were kept in Dulbecco’s Modified Eagle medium (DMEM) with Glutamax-l containing 4.5 g/L D-Glucose, pyruvate, 1% Penicillin/Streptomycin and 10% fetal calf serum. HAP1 cells were cultured in Iscove’s Modified Dulbecco’s Medium (IMDM) containing 10% fetal calf serum and 1% Penicillin/Streptomycin (P/S). DRG were collected and primary sensory neurons were cultured as described.^82^

### Method details

#### Drug administration

NAD^+^ precursor, nicotinamide riboside (NR) was injected intraperitoneal (500 mg/kg, Tebu-bio)^83^ or intrathecal. In the CFA experiment, we measured hyperalgesia 4 h after injection of NR. Intrathecal injections (5 μL) with NR (50 μg), myxothiazol (50 μM, Sigma-Aldrich), mito-tempol^34^ (25 μg, Sanbio) and oligodeoxynucleotide (3 μg/μL day 4, 5 and 6 after carrageenan, Sigma-Aldrich), were performed under light isoflurane anesthesia as described.^16^^,^^84^ The following phosphorothioated sequences were used to specifically target mouse ATPSc-KMT:

*ATPSCKMT*: CCCGCCTGTCTTTCTTCCTC *MM*: CGCCTCCGTTCCTTTCTCCT.

#### DNA and viral constructs

ATPSCKMT (NM_199133.4) was cloned in pIRES2-AcGFP1 (Clontech). *ATPSCKMT* overexpression was achieved, using Lipofectamine 2000 (Life technology) according to manufacturer’s instructions.

We generated a bicistronic herpes simplex virus (HSV) construct expressing ATPsc-KMT and GFP as described previously.^16^ Control empty HSV (HSV-EV) only expressed GFP. HSV was produced as previously described.^79^ Mice were inoculated twice (day −3 and day −1 prior to PGE_2_) intraplantar with 2.5 μL of 1.4 × 10^7^ pfu/mL.

Twenty-four hours after plating, sensory neuron cultures were inoculated with HSV (MOI of 2, 10,000 pfu) for 3 days. The anti-mitotic fluoro-deoxyuridine (FDU 13.3 μg/mL, Sigma-Aldrich) was added to inhibit satellite glia cell growth in the neuronal cultures.

Lentivirus expressing shRNA against mouse *ATPSCKMT* was produced according protocol (Sigma-Aldrich). In short, 2x10^6^ HEK293T cells were cultured in Ø10 cm Petri dish and transfected with PEI and a mix of plasmids, containing 5 μg MISSION shRNA *ATPSCKMT* (or scrambled control)*,* 1.8 μg envelope vector (pMD2.G) and 1.8 μg packaging vector (psPAX2). The medium was replaced the following day. Subsequently, 48 and 72 h after transfection, medium containing lentivirus was collected for experiments. For *ATPSCKMT* knockdown, primary sensory neurons were incubated with lentivirus (50% of the medium) for 2 days.

#### Mitochondrial superoxide detection with immunofluorescence

*In vivo*, MitoTrackerRedCM-H2XROS, which emits fluorescence upon oxidation, to measure mitochondrial superoxide production (10 μL of 100 μM, Life technology)^16^^,^^32^ was injected intrathecal at day 7 after intraplantar carrageenan administration. Six hours later, mice were perfused with PBS and 4% PFA and DRG were collected. Tissues were cryoprotected in sucrose, embedded in OCT compound (Sakura), and frozen at −80°C. Cryosections (10 μm) of lumbar DRG were stained with Neurotrace 435/455 (1:300, ThermoFisher) to visualize the neurons. For mitochondrial superoxide production measurements *in vitro*, HAP1 cells were incubated with 200 nM MitoTrackerRedCM-H2XROS in HBSS for 30 min. After PBS washes cells were fixed with 4% PFA. Fluorescence was captured using Olympus IX83 microscope and analyzed with ImageJ software. The number of positive MitoTrackerRedCM-H2XROS neurons was determined by the amount of cells that had a higher fluorescence than the mean + 2x SD of the naive group.

#### Flow cytometry analysis for measuring mitochondrial mass

DRG (L3–L5) were collected from non-primed and primed mice. Tissues were gently minced and digested at 37 °C for 25 min with an enzyme cocktail (5 mg collagenase type I with 2.5 mg trypsin, Sigma-Aldrich) in 5 mL DMEM. Cells were incubated with 100 nM nonyl acridine orange (NAO, Enzo LifeSciences) for 30 min at 37°C to detect mitochondrial mass. After washing with PBS, the cells were incubated with ef506 viability marker (1:1000, eBioscience) for 20 min at 4°C, followed by a CD45-APC staining (1:600, eBioscience) for 20 min at 4°C, to exclude the immune cells. Samples were acquired by Canto II flow cytometer (BD Biosciences) and analyzed with FACSDIVA software.

#### Mitochondrial bioenergetics assessment

N2A cells were plated (12.500 cells/well) on poly-*l*-lysine-coated XF24 wells plates (Seahorse Bioscience) in DMEM (high glucose, with pyruvate) (Gibco) + 10% FCS +1% P/S. Primary DRG neurons (15K) were seeded on poly-*d*-ornithine/laminin coated XF24 wells plate, grown overnight at 37°C and transduced with HSV or lentivirus (see above). The cells were washed and placed in Seahorse XF-assay media (pH 7.4) containing 25 mM glucose, 4 mM glutamine and 1 mM pyruvate at 37°C for 1 h. The Seahorse Bioscience XFe24 Analyzer (Seahorse Bioscience) was used to measure oxygen consumption rate (OCR). After assessing basal OCR, 2 μM oligomycin, 2 μM FCCP and 2 μM of rotenone and antimycin A (all from Sigma-Aldrich) were injected after cycle 3, 6, and 9 respectively. Each cycle consisted of 1.5 min of mixing, 2 min waiting, and 3 min of measurements. For each condition three cycles were used. Glycolytic capacity was measured by subtracting the extracellular acidification rates after addition of oligomycin from basal ECAR levels. The measurements were normalized for protein content.

Mitochondria from N2A cells expressing ATPsC-KMT or control EV were isolated according to Iuso et al.,.^85^ To measure complex II driven respiration, 5 μg of mitochondria were added in a non-coated XF24 plates in MAS buffer (220 mM d-Mannitol, 70 mM sucrose, 10 mM KH2PO_4_, 5 mM MgCl_2_, 2 mM HEPES, 1 mM EGTA, and 0.2% (w/v) of fatty acid-free BSA, pH 7.2) supplemented with 10 mM succinate and 2 μM rotenone. OCR levels were measured under basal conditions, and after sequential addition of ADP (2 mM), oligomycin (3.2 μM), FCCP (4 μM), and antimycin A (4 μM), to measure state III. Each assay cycle consisted of 1 min of mixing and 3 min of OCR measurements. For each condition, three cycles were used to determine the average OCR under given condition.

Measurement of mitochondrial respiration in sciatic nerves was performed according to Krukowski et al.,.^86^ In short, sciatic nerves were isolated and stored for maximum 1 h on ice in XF medium, until all nerves were collected. Sciatic nerves were transferred into islet capture XF24 microplates containing XF-assay media supplemented with 5.5 mM glucose, 0.5 mM sodium pyruvate, and 1 mM glutamine (pH 7.4). Plates were incubated in a non-CO_2_ incubator to degas for 2 h at 37°C. OCR levels were measured under basal conditions and after sequential addition of oligomycin (12 μM), FCCP (20 μM), and antimycin A/rotenone (20 μM). Each assay cycle consisted of 3 min of mixing, 3 min waiting, and 4 min of measurements. For each condition four cycles were used. The measured OCR was normalized for protein content.

N2A cells were used to measure complex-specific respiration. To assess complex I activity, cells were incubated with pyruvate and malate. To assess complex II/III activity, cells were incubated with succinate and rotenone to supply electrons to the ETC via complex II and rotenone to inhibit complex I. To assess complex IV activity, cells were incubated with N,N,N′,N′-tetramethyl-*p*-phenylenediamine (TMPD) ascorbate, to supply electrons to the ETC via complex IV, and antimycin A to inhibit complex II/III. All reagent solutions were prepared in MAS buffer in Ultrapure or tissue-culture-grade H2O and adjusted to pH 7.2 at 37°C, unless stated otherwise. Ascorbate (1.33 M, Genfarma, Toledo, Spain), TMPD (10 mM, Sigma-Aldrich) dissolved in 10 mM ascorbate and ADP (50 mM, Sigma-Aldrich) were freshly prepared. All other reagents (Sigma-Aldrich) were prepared beforehand and stored as stock solutions at −20°C, including pyruvate (1 M), succinate (0.5 M), malate (0.5 M), dichloroacetic acid (1 M; DCA), rotenone (2.5 mM), antimycin A (2.5 mM), myxothiazol (10 mM) and carbonyl cyanide-*p*-trifluoromethoxyphenylhydrazone (2.5 mM; FCCP). The medium was removed and cells were washed once with MAS buffer. MAS buffer containing 10 mM pyruvate, 10 mM malate, 2 mM DCA, 4 mM ADP, 1 nM XF PMP (Seahorse Bioscience) and with 2.5 mM FCCP and 2.5 mM oligomycin was added to the cells. The cartridge was calibrated one day prior to the experiment following manufacturer’s instructions. The cartridge was loaded with 75 μL of MAS buffer containing the following compounds: (port A) rotenone (2 μM), (port B) succinate (10 mM) and rotenone (2 μM), (port C) antimycin A (2 μM) and (port D) ascorbate (10 mM), TMPD (100 μM) and antimycin A (2 μM). The OCR was measured using the following steps: mix/delay/measure times were 0.5 min/0.5 min/2 min, an equilibration step was not included and three measurements were made for each step. OCR was recorded as pM/minute.

#### Patch clamp electrophysiology and calcium imaging

DRG neurons of *Atpsckmt*^*+/−*^ and WT (*Atpsckmt*^*+/+*^) littermates control mice were cultured as described previously.^82^ Cells were seeded on poly-L-lysine (0.01 mg/mL; Sigma) and laminin (0.02 mg/mL; Sigma)-coated 35-mm dishes and cultured in a 5% CO2 incubator at 37°C. Cells were used the following 1 to 2 days.

Action potentials were recorded in a whole cell patch clamp experiment. Cells were bathed in solution at room temperature, containing (in mM): NaCl 120, KCl 2.5, CaCl_2_ 2.5, MgCl_2_ 1.3, NaHCO_3_ 17.5, HEPES 10, glucose 10, pH 7.40/NaOH. Pipettes had a resistance of 3–4 MΩ when filled with a solution containing (in mmol/L): KCl 10, K-Gluconate 125, CaCl_2_ 0.6, MgCl_2_ 2, HEPES 5, Na_2_ATP 4, EGTA 5, pH 7.20/KOH. Recordings were made using a HEKA EPC 10 Single USB amplifier controlled by HEKA Patchmaster 2x90.2.

For calcium imaging, changes in the capsaicin-evoked calcium response were measured by loading cells with 5 μM Fura-2-AM (Invitrogen) for 20 min in 140 mM NaCl, 4 mM KCl, 1 mM MgCl_2_, 2 mM CaCl_2_, 10mM HEPES, and 10mM Glucose; pH 7.3/NaOH. Cells were excited at 340 and 380 nm wavelengths and fluorescence was measured every 3 s at 510 nm using Olympus IX83 inverted microscope (10x objective). The ratio 340/380 is directly correlated with the amount of intracellular calcium. Recordings were performed as previously described.^87^ Briefly, every experiment included a 5 min baseline measurement followed by a stimulation of the cells by superfusion with capsaicin (0.03 μM, Sigma Aldrich) for 21 s followed by superfusion of medium. A subsequent 5 min of superfusion with high K^+^-buffer (4mM NaCl, 140mM KCl, 1mM MgCl_2_, 2mM CaCl_2_, 10mM HEPES, and 10mM Glucose; pH 7.4/NaOH) was added at the end of each experiment to depolarize the neurons to confirm cell viability and functionality.

#### Immunostaining

DRG were collected and directly embedded in OCT compound (Sakura) and frozen at −80°C. Cryosections (10 μm) of lumbar DRG were post-fixed in PFA and stained with anti-ATPSc-KMT (1:500, biorbyt) or ATF3(1:1000, ThermoFisher). ATPSc-KMT or ATF3 were visualized by using Alexa Fluor 594 or 488-conjugated secondary antibody. Neurons were visualized with Neurotrace 435/455 or 640/660 (1:300, ThermoFisher). Photographs were captured using an Olympus IX83 microscope using identical exposure times for all slides within one experiment. Fluorescence intensity was analyzed with ImageJ software. Fluorescence was analyzed in small diameter neurons <20 μm and medium/large diameter neurons >20 μm^33^

#### Collection of cell lysates for metabolomics

HAP1 cells expressing ATPSc-KMT (WT), ATPSc-KMT^−/−^ HAP1 cells, as well as, ATPSc-KMT^−/−^ cells reconstituted with WT or E117A mutated ATPSc-KMT were plated in 6-well plates and cultured for 48 h. Medium was refreshed 24 h after plating. Cell collection was done by washing cells with cold PBS (4°C), followed by cell scraping in 0.5 mL ice-cold methanol. Next, methanol samples were transferred into Eppendorf tubes, centrifuged (13000 rpm for 5 min at 4°C), and then supernatants were transferred to new 1.5-mL Eppendorf tubes. The samples were evaporated at 40°C under a gentle stream of nitrogen until complete dryness, and reconstituted with 500 μL of UPLC-grade methanol (room temperature). The reconstituted samples were stored at −80°C until analysis was performed.

#### Non-quantitative direct-infusion high-resolution mass spectrometry (DI-HRMS)

A non-quantitative DI-HRMS metabolomics method was used as previously described (Haijes et al. 2019). Samples were analyzed using a TriVersa NanoMate system (Advion, Ithaca, NY, USA) controlled by Chipsoft software (version 8.3.3, Advion). Data acquisition was performed using Xcalibur software (version 3.0, Thermo Scientific, Waltham,MA, USA). Di-hrms is unable to separate isomers, therefore mass peak intensities consisted of summed intensities of these isomers. Metabolite annotation was performed using a peak calling pipeline developed in R programming language, annotated the raw mass spectrometry data according to the Human Metabolome DataBase (HMDB) allowing for 2 ppm deviation from the theoretical m/z. This resulted in ∼3800 metabolite annotations corresponding to ∼1900 unique metabolite features. Data was analyzed with MetaboAnalyst 5.0. In case of multiple possible annotations per feature, isobaric compounds were processed as only one metabolite for statistical purposes.

#### Ultra-high performance liquid chromatography (UPLC)

Liquid chromatography coupled to tandem mass spectrometry (LC/MS-MS) was performed using a Q Exactive HF hybrid quadrupole-Orbitrap mass spectrometer (Thermo Fisher Scientific). In short, cells from a single well of a 6-wells plate were quenched using 250 μL of −80°C 80:20 methanol/water. Next, cells were incubated on dry ice for 20 min and scraped on ice, subsequently, the extract was collected in 1.5 mL Eppendorf tubes. The Eppendorf’s containing the extract were then vortexed and centrifuged at 14000 G, after that the supernatant was collected in a new Eppendorf tube. Extracts were dried under a flow of nitrogen at 40°C, reconstituted in 40 μL of water after which 10 μL of 100 μM β-NAD-d4 (Toronto Research Chemicals) was added resulting in a final concentration of 20μM for the internal standard (IS). Prior to analysis, calibration samples were prepared by dilution and subsequent addition of the internal standard. To this end, standards were serially diluted in a range of 125 μM–3.9 μM (NAD^+)^ and 15.5 μM–1 μM (NADH). Sample extracts and standards were transferred to 12 × 32mm glass screw neck vials (Waters) and then injected onto a Sunshell RP-Aqua column(150 mm × 3 mm i.d., 2.6 μm; ChromaNik Technologies Inc., Osaka, Japan). For this purpose, a binary solvent gradient comprising of 0.1% formic acid in water (Mobile phase A) and 0.1% formic acid in acetonitrile (Mobile phase B) was used. The flow rate was 0.6 mL/min with the following gradient elution: isocratic 100% A from 0–0.5 min, linear from 100 to 85% A from 0.5–2 min, linear from 85 to 75% A from 2–2.75 min, linear from 75 to 30% A from 2.75–3.5 min, isocratic 30% A from 3.5–7 min, linear from 30 to 100% A from 7–7.2 min and isocratic 100% A (initial solvent conditions) from 7.2 to 12 to equilibrate the column. The column flow was then directed into the MS detector and the samples were measured. Raw data integration and inspection were performed using TraceFinder 4.1 software (Thermo Fisher Scientific).

#### Quantification TCA cycle intermediates

The quantification of TCA cycle intermediates in cell lysates was performed on a Waters Acquity ultra performance liquid chromatography system (Waters Corp., Milford, USA), as described by Broeks et al.^88^ The assay was performed for technical triplicates of cell lysates and data were corrected for total protein concentration.

#### Western Blot

Protein concentrations of the total cell lysates of lumbar DRG were determined using a Bradford assay (Bio-Rad). Protein samples (40 μg) were separated by 4–10% SDS-PAGE and transferred to a PVDF membrane (Immobilon-P, Millipore). Membrane was stained with anti-OXPHOS (1:1000, ThermoFisher scientific) or goat anti-β-actin (1:1000, abcam), followed by incubation with 1:5000 donkey anti goat-HRP (abcam). Specific bands were visualized by chemiluminescence (ECL, Advansta) and imaging system Biorad.

#### Real-time (RT)-PCR

Total RNA from freshly isolated DRG or hind paws was isolated using TRizol and RNeasy mini kit (Qiagen). cDNA was synthesized using iScript reverse transcription supermix, according to manufacture protocol (Bio-Rad, Hercules, CA). Quantitative real-time PCR reaction was performed with a QuantStudio 3 (ThermoFisher) following manufacturer’s instructions. We used the following primers.

**Table:** 

Target	Forward	Reverse
*ATPSCKMT*	TggTgTgCCCCAgATgAT	TgCCCTCTCCAgTggTgT
*F4/80* *IL1β*	TTACgATggAATTCTCCTTgTATATCA CAACCAACAAgTgATATTCT	CACAgCAggAAggTggCTATg gATCCACACTCTCCAgCTgCA
*IL6*	TCTAATTCATATCTTCAACCAAgAgg	TggTCCTTAgCCACTCCTTC
*TBP*	CCTTgTACCCTTCACCAATgAC	ACAgCCAAgATTCACggTAgA
*Rictor*	TgCgATATTggCCATAgTgA	ACCCggCTgCTCTTACTTCT
*18S*	gTAACCCgTTgAACCCCATT	CCATCCAATCggTAgTAgCg
*Β-actin*	AgAgggAAATCgTgCgTgAC	CAATAgTgATgACCTggCCgT
*HPRT*	TCCTCCTCAgACCgCTTTT	CCTggTTCATCATCgCTAATC
*YWHAZ*	TAggTCATCgTggAgggTCg	gAAgCATTggggATCAAgAACTT

The most stable housekeeping genes per tissue were determined by measuring multiple internal controls (*β-actin, 18S, GAPDH, B2M, HRPT, β-tubulin, Rictor, TBP, YWHAZ*) and were analyzed with geNorm.^89^ mRNA expression is represented as relative expression = 2^Ct(average of reference genes)−Ct(target).^ For mRNA expression in the paws, we used the average Ct values of *HPRT*, *β-actin* and *YWHAZ* as reference; for mRNA expression in DRG we used the average of *18S*, *TBP* and *Rictor* as reference.

#### Preparation of DRG extract enriched for mitochondrial inner membrane proteins

Preparation of DRG extract enriched for mitochondrial inner membrane proteins was performed at 4°C, similarly as described previously.^17^ DRG, frozen in PBS, were thawed on ice, centrifuged (11000 x g, 1 min), and the PBS was discarded. The pellet was resuspended in 50 μL PBS, supplemented with 0.5 mg/mL digitonin and 1% protease inhibitor cocktail (P8340), and incubated for 5 min on ice. The suspension was centrifuged (11000 x g, 10 min) and the supernatant discarded. The pellet was resuspended in 50 μL of Extraction Buffer (50 mM Tris-HCl pH 7.4, 100 mM NaCl, 1% *n*-dedecyl-β-D-maltoside, 5% glycerol, and protease inhibitors), and incubated on ice for 5 min. The suspension was centrifuged (16100 x g, 5 min) and *n*-dedecyl-β-D-maltoside-extracted proteins were recovered in the supernatant.

#### Mass spectrometry analysis of ATPSc from DRG

Proteins in DRG extracts were resolved by SDS-PAGE, stained with Coomassie, and the region of gel containing ATPSc, i.e., around the 8 kDa marker, was excised and subjected to in-gel chymotrypsin (Roche) digestion. The resulting proteolytic fragments were analyzed by liquid chromatography MS, similarly as described previously.^90^ MS data were analyzed using an in-house maintained human protein sequence databases using SEQUEST and Proteome Discoverer (Thermo Fisher Scientific). The mass tolerances of a fragment ion and a parent ion were set as 0.5 Da and 10 ppm, respectively. Methionine oxidation, cysteine carbamido-methylation, lysine mono-, di- and trimethylation, and arginine mono- and dimethylation were selected as variable modifications. MS/MS spectra of peptides corresponding to methylated Lys-43 in ATPSc were manually searched by Qual Browser (v2.0.7).

### Quantification and statistical analysis

All data are presented as mean ± SD and were analyzed with GraphPad Prism version 9.3 using Student’s *t* test (two-tailed, unpaired), Fisher-exact test, one-way, two-way ANOVA, or two-way ANOVA with repeated measures as appropriate, followed by post-hoc analysis. The used post-hoc analyses are indicated in each figure. A p value less than 0.05 was considered statistically significant and each significance is indicated with ∗p < 0.05, ∗∗p < 0.01, ∗∗∗p < 0.001. The n is depicted in the figures, or in the figure legends.

## Supplemental information

Document S1. Figures S1–S5

Document S2. Article plus supplemental information
