# Supplementary material for: Inflammation-induced mitochondrial and metabolic disturbances in sensory neurons control the switch from acute to chronic pain
Source: Cell Rep Med. 2023 Nov 8;4(11):101265. doi: 10.1016/j.xcrm.2023.101265 (PMC10694662; doi:10.1016/j.xcrm.2023.101265)
Supplement: Document S1. Figures S1–S5 [file mmc1.pdf]

**Cell Reports Medicine, Volume *4***

## Supplemental information

# Inflammation-induced mitochondrial and metabolic disturbances in sensory neurons control the switch from acute to chronic pain

**Hanneke L.D.M. Willemen, Patrícia Silva Santos Ribeiro, Melissa Broeks, Nils Meijer, Sabine Versteeg, Annefien Tiggeler, Teun P. de Boer, Jędrzej M. Małecki, Pål Ø. Falnes, Judith Jans, and Niels Eijkelkamp**

## Supplementary figures:

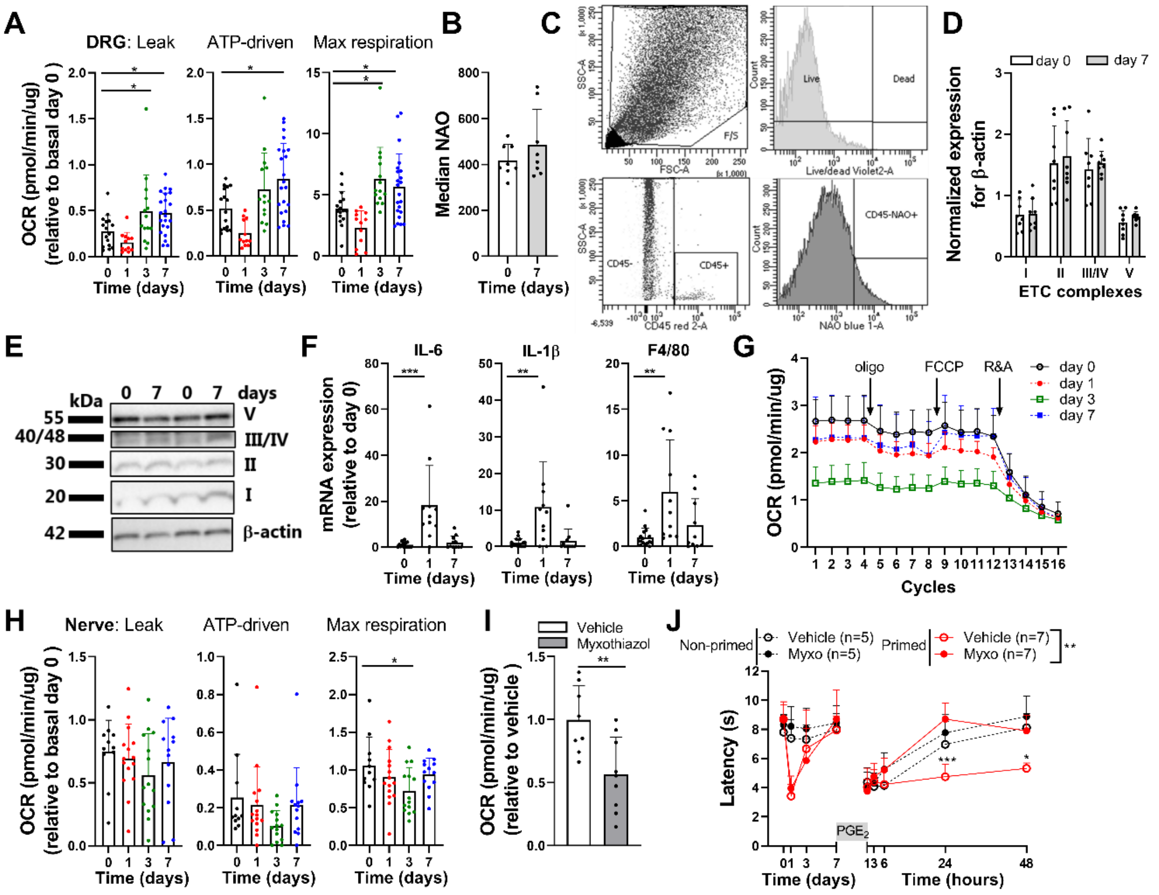

**Figure S1: A priming stimulus alters mitochondrial activity in DRG neurons and impairs resolution of PGE2-induced hyperalgesia. Related to Figure 1.**

**A)** OCR in DRG neuron cultures at day 0 (n=16), 1 (n=12), 3 (n=14) and 7 (n=22) after intraplantar carrageenan injection. Leak, ATP-driven and maximal respiration was measured after sequential addition of oligomycin (ATP synthase inhibitor), carbonyl cyanide-p-trifluoromethoxyphenylhydrazone (FCCP, uncoupling protonophore that dissipates mitochondrial membrane potential), and a mixture of rotenone (inhibitor of Complex I) and antimycin A (inhibitor of Complex III). **B)** FACS quantification of nonyl acridine orange (NAO) positive CD45- cells in DRG neurons, as a measure of mitochondrial content in non-primed (day 0) and primed (day 7) mice (n=8). **C)** FACS gating strategy for measuring mitochondrial mass in B. **D)** Quantified expression of the five different OXPHOS complexes (normalized for *β-actin*) in DRG of primed (day 7) and non-primed mice (day 0) mice (n=8). **E** ) Exemplar image of the western blot that was used to quantify expression for D. **F)** Relative mRNA expression of inflammatory markers in the paw at indicated days after intraplantar carrageenan injection (n=6). **G)** OCR was measured over time in sciatic nerves at day 0 (n=11), 1 (n=15), 3 (n=14) and 7 (n=13) after intraplantar carrageenan injection. **H)** Leak, ATP-driven and maximal respiration in sciatic nerves was measured, similar as in B. **I)** Basal OCR in DRG neuron cultures of primed mice (day 7) 1 hour after intrathecal injection of vehicle or myxothiazol (myxo, 50 µM) (n=8). **J)** Course of $PGE_2$-induced thermal hyperalgesia after intrathecal injection of vehicle or myxothiazol (myxo, 50 µM) at day 7 in carrageenan-primed

and non-primed mice, and 15 min prior to intraplantar $PGE_2$. Data are represented as mean ± SD. *P < 0.05, **P < 0.01, ***P < 0.001. Statistical analyses were performed by student's T-test (B and I), one-way ANOVA (A, F, H) followed by Dunnett's multiple comparison test or two-way ANOVA followed by a post-hoc Sidak's multiple comparison test (D) and with repeated measures (J: stars indicate significance comparing primed conditions).

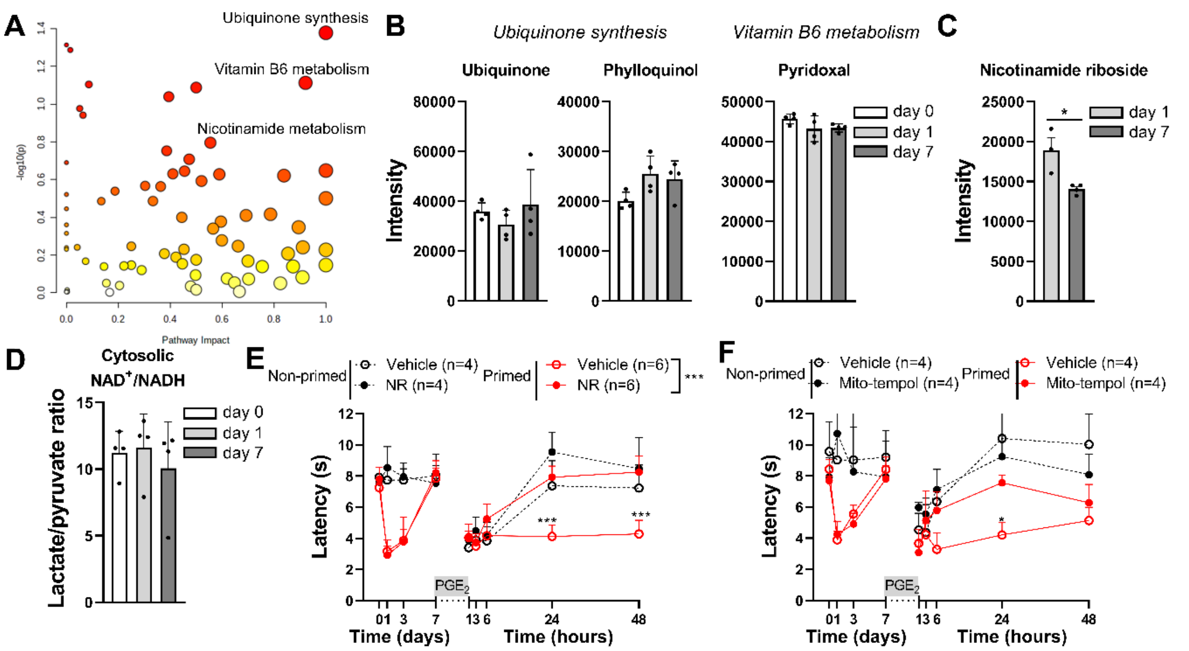


**Figure S2: $NAD^+$ pathway is affected after priming and $NAD^+$ supplementation or attenuation of oxidative stress restores resolution of $PGE_2$-induced hyperalgesia in primed mice. Related to Figure 2.**

**A)** Pathway analysis of metabolites detected in lumbar DRG of untreated mice (day 0) versus DRG from primed mice (day 7 after carrageenan injection). Colors varying from yellow to red mean different levels of significance, with the most significantly affected pathways indicated in red. Figure created with MetaboAnalyst 5.0. **B/C)** Intensity of indicated metabolites in lumbar DRG at indicated days (n=4) and involved in **B)** ubiquinone synthesis and vitamin B6 metabolism, and **C)** nicotinamide riboside. **D)** Lactate/pyruvate ratio as an indirect measure of cytosolic $NAD^+/NADH$ ratio (n=4). **E/F)** Course of $PGE_2$-induced thermal hyperalgesia after **E)** intraperitoneal injection with nicotinamide riboside (NR, 500 mg/kg) or **F)** intrathecal administration of mito-tempol (25 ug) at day 7 in carrageenan-primed and non-primed mice and 15 min prior to intraplantar $PGE_2$. Data are represented as mean ± SD. *P < 0.05, ***P < 0.001. Statistical analyses were performed by, student's T-test (C), one-way ANOVA (B and D) followed by Dunnett's multiple comparison test or two-way repeated measures ANOVA followed by a post-hoc Sidak's multiple comparison test (E and F: stars indicate significance comparing primed conditions).

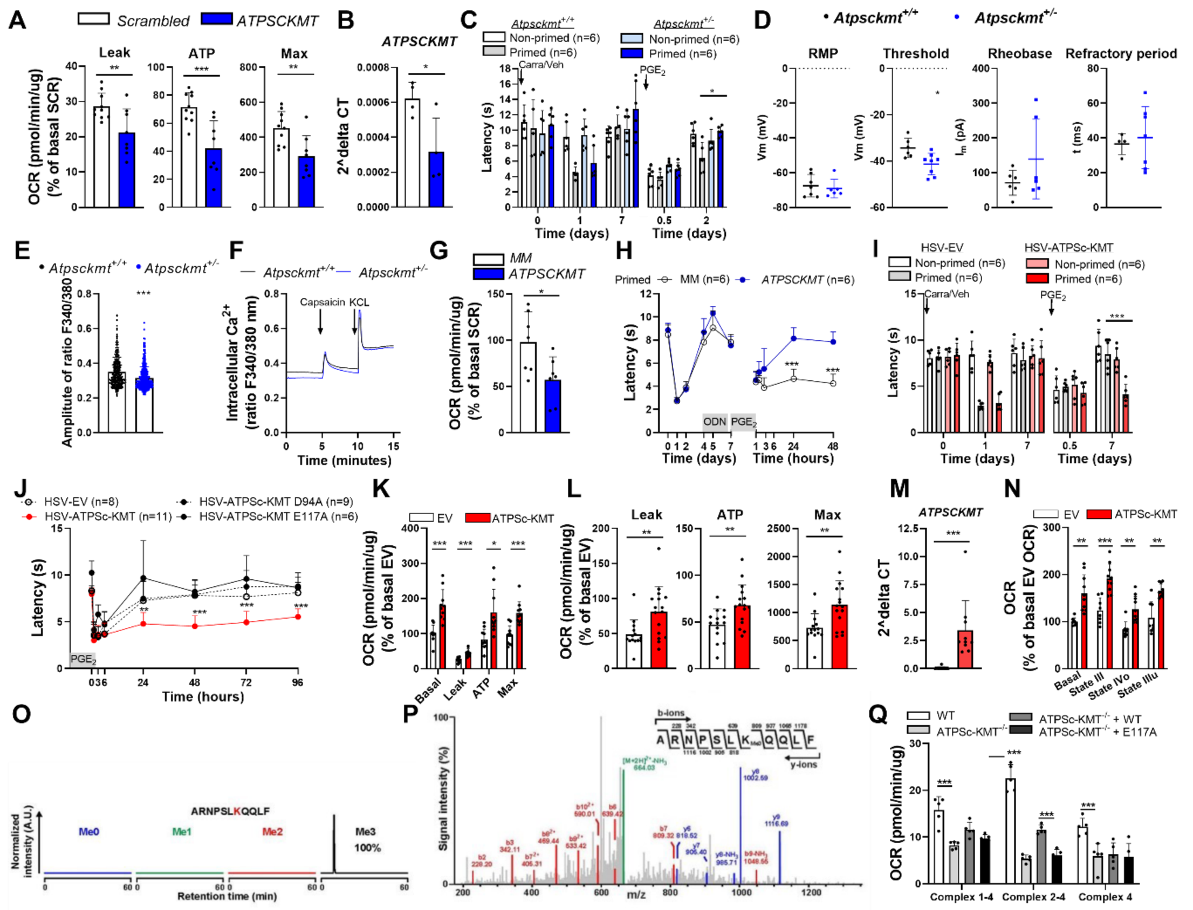


**Figure S3: ATPSc-KMT expression promotes mitochondrial hyperactivity in DRG neurons and mimics hyperalgesic priming. Related to Figure 3.**

**A)** OCR measurements in DRG neuron cultures after lentiviral-mediated *ATPSCKMT* knockdown (n=8) compared to scrambled-controls (n=10). Leak, ATP-driven and maximal respiration was measured after sequential addition of oligomycin, FCCP, and mixture of rotenone and antimycin A. **B)** *ATPSCKMT* mRNA expression in DRG neurons after lentiviral-mediated knockdown compared to scrambled-controls (n=4). **C)** Course of $PGE_2$-induced thermal hyperalgesia in carrageenan-primed and non-primed Atpsckmt$^{+/+}$ (WT) and Atpsckmt$^{+/-}$ mice. **D)** Resting membrane potential (RMP), threshold potential, rheobase and relative refractory period measured with patch-clamp electrophysiology in DRG neurons of heterozygous *Atpsckmt*$^{+/-}$ mice and WT littermates (*Atpsckmt*$^{+/+}$). **E/F)** Total calcium fluxes were quantified **(E)** at baseline and 3 nM capsaicin-evoked $Ca^{2+}$ responses were measured in cultured DRG neurons of *Atpsckmt*$^{+/-}$ (n= 514 neurons) and *Atpsckmt*$^{+/+}$ mice (n= 325 neurons). 140 mM KCL was added at the end of each experiment to depolarize the neurons to confirm cell viability and functionality. **G)** Basal OCR measurements in DRG neuron cultures at day 7 and after intrathecal injection of ATPSCKMT antisense or mismatched control (MM) at day 4, 5 and 6 (n=7). **H)** Course of $PGE_2$-induced thermal hyperalgesia after intrathecal *ATPSCKMT*-antisense or MM oligodeoxynucleotide (ODN, 3 µg/µl, 5 µL) at day 4, 5 and 6 in carrageenan-primed mice. **I/J)** Course of $PGE_2$-induced thermal hyperalgesia (**I**) after intrathecal *ATPSCKMT*-antisense injections (day 4, 5 and 6) in primed and non-primed mice. To reconstitute *ATPSCKMT* expression, mice received intraplantar injections with HSV-ATPSc-KMT (35.000 pfu/paw) or HSV-EV as control at day 4 and 6 after, **J)** in mice expressing ATPSc-KMT, its

catalytically-inactive mutant (D94A or E117A), or control empty vector (EV) in DRG neurons. Intraplantar HSV injections were administrated at day -3 and -1 (35.000 pfu/paw). **K/L)** OCR measurements in **K)** N2A's after overexpression of ATPSc-KMT or EV control (n=10), and **L)** primary sensory neurons after HSV-mediated expression of ATPSc-KMT (n=16) or EV (n=14); measurements were performed as described in A. **M)** *ATPSCKMT* mRNA expression in DRG neurons after transduction with HSV (n=10). **N)** OCR measurements in isolated mitochondria from N2A cells overexpressing ATPSc-KMT (n=10) or EV (n=8). Complex-II driven basal, State III, State IVo and State IIIu respiration was measured after sequential addition of ADP, oligomycin, FCCP and antimycin A. **O)** Methylation state of ARNPSL**K**QQLF peptide containing lysine-43 (bold) of the ATP synthase c-subunit in DRG under naïve conditions. **P)** MS/MS fragmentation spectrum showing trimethylation of lysine-43 in ARNPSLK(me3)QQLF in DRG. **Q)** OCR measurements in permeabilized HAP1 cells in presence of FCCP/Oligomycin (n=5). Cells were incubated with pyruvate and malate to supply electrons to the ETC via complex I, succinate and rotenone to supply electrons to the ETC via complex II and TMPD, ascorbate and antimycin A to supply electrons to the ETC via complex IV. Data are represented as mean ± SD. *P < 0.05, **P < 0.01, ***P < 0.001. Statistical analyses were performed by Student's t-test (A-/B, D/E, G, L, M), one-way ANOVA (K and N) followed by Dunnett's multiple comparison test or two-way repeated measures ANOVA followed by a post-hoc Sidak's multiple comparison test (C, H, I, J and Q: stars indicate significance compared to ATPSc-KMT-primed conditions). Primed mice with ATPSc-KMT overexpression are indicated with red bars/lines and blue bars/lines indicate *ATPSCKMT* knockdown.

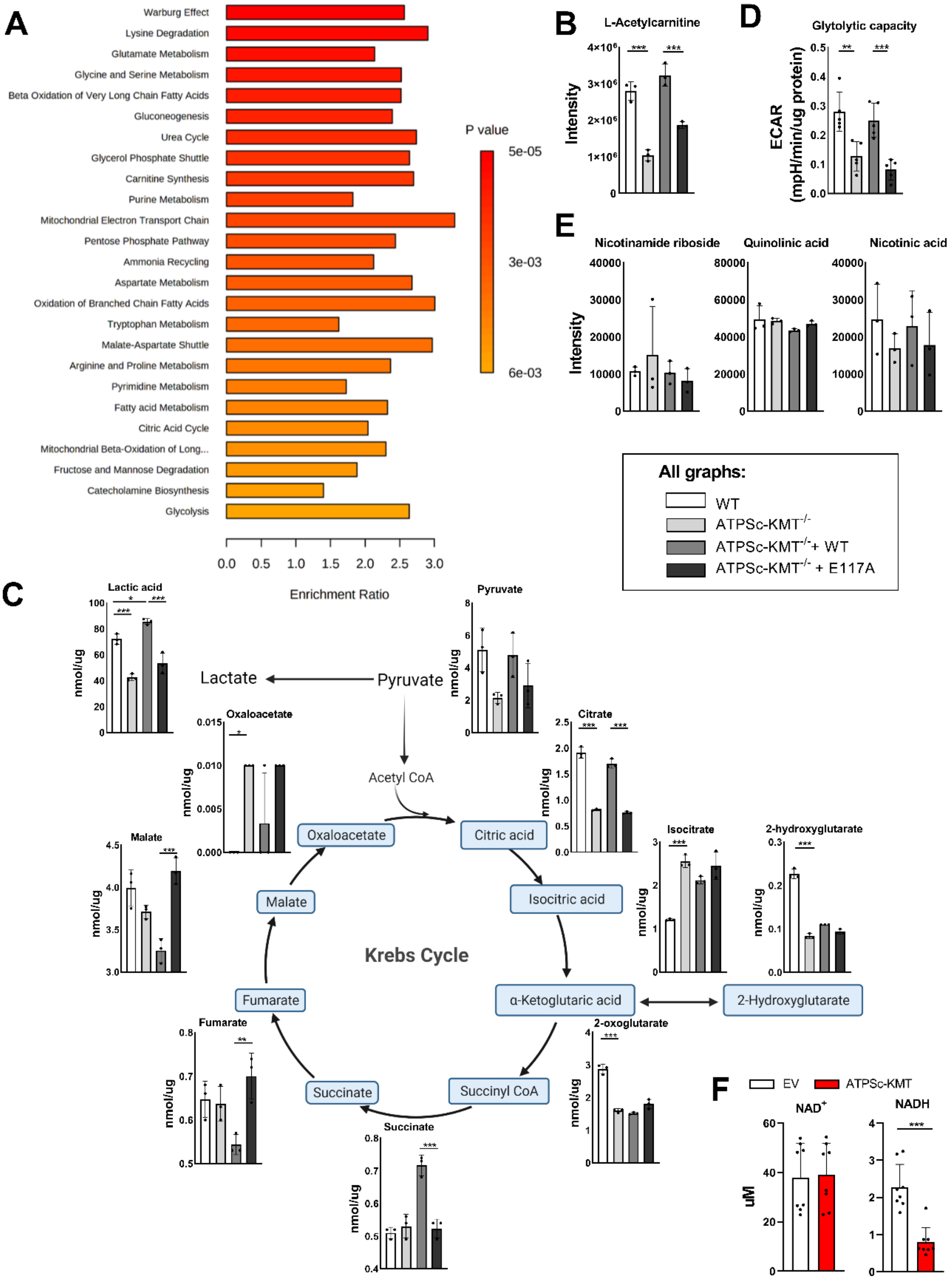


**Figure S4**: **ATPSc-KMT affects cellular metabolism and redox balance. Related to Figure 4.**

**A)** List of top 25 pathways/processes significantly changed between HAP1 WT and ATPSc-KMT KO cells (n=3). **B)** An example of a functionally related metabolite that is significantly changed between ATPSc-KMT-proficient HAP1 cells (WT and KO cells reconstituted with WT ATPSc-KMT) or ATPSc-KMT-deficient HAP1 cells (KO and KO cells reconstituted with ATPSc-KMT E117A mutant) (n=3). **C)** Comparison of metabolite

levels in ATPSc-KMT-proficient and –deficient HAP1 cells. Targeted screen was performed with focus on Krebs (TCA) cycle metabolites; a-ketoglutaric acid also referred as 2-oxoglutarate (n=3). Figure created with BioRender.com **D)** ECAR, as measure for glycolytic capacity, in ATPSc-KMT-proficient and –deficient HAP1 cells (n=5). **E)** Intensity of metabolites involved in generation of $NAD^+$ (n=3) in ATPSc-KMT-proficient and –deficient HAP1 cells. **F)** $NAD^+$ and NADH pool in N2A cells after overexpression of ATPSc-KMT or EV control (n=8). Data are represented as mean ± SD. *P < 0.05, **P < 0.01, ***P < 0.001. Statistical analyses were performed by Student's t-test (F) or one-way ANOVA (B-E) followed by Dunnett's multiple comparison test.

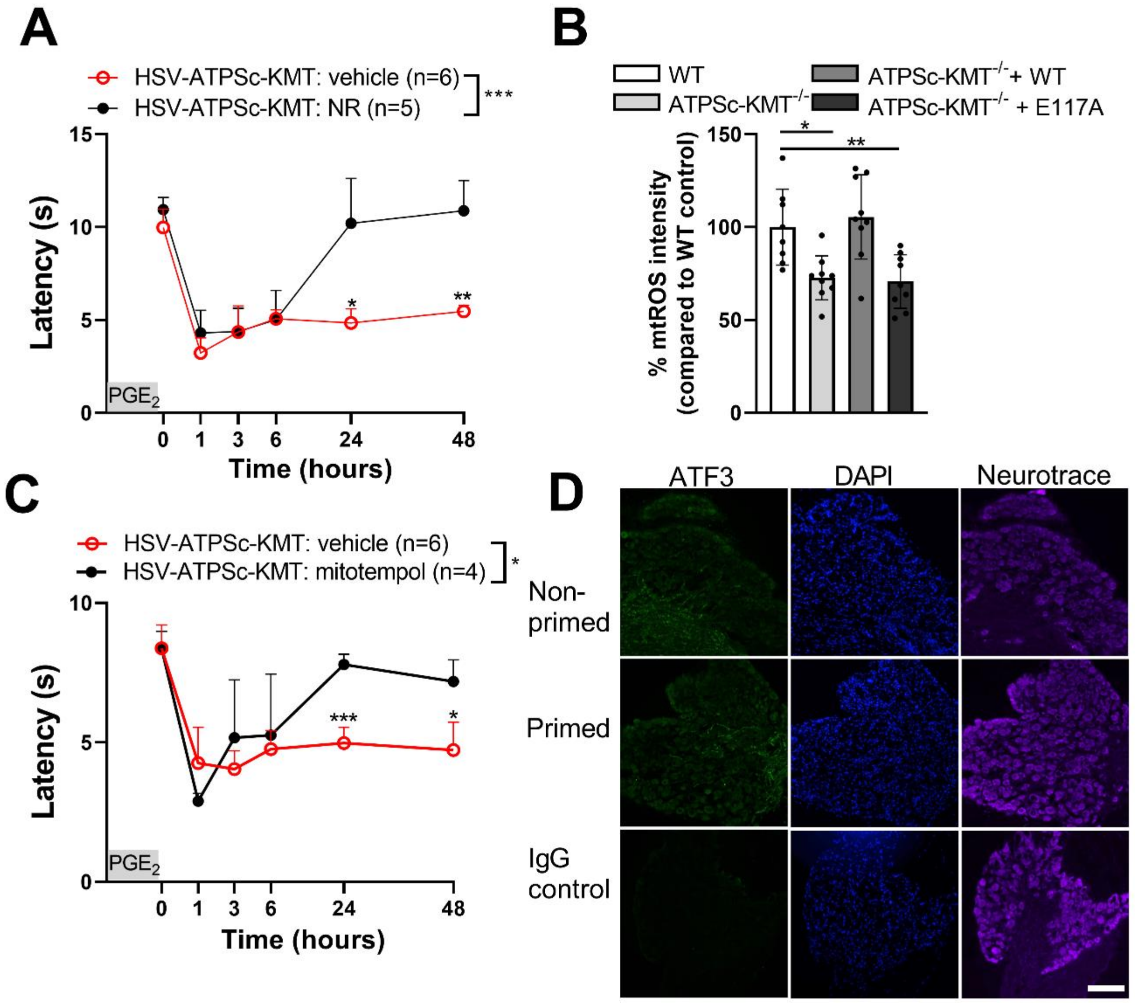


**Figure S5**: **Blocking oxidative stress or NAD+ supplementation restores the resolution of $PGE_2$-induced hyperalgesia in mice expressing HSV-ATPSc-KMT. Related to Figure 5.**

**A)** Course of $PGE_2$-induced thermal hyperalgesia after intraperitoneal injection with nicotinamide riboside (NR, 500 mg/kg) in mice expressing ATPSc-KMT in DRG neurons. Intraplantar HSV injections were administrated at day -3 and -1 (35.000 pfu/paw). **B)** mtROS formation in ATPSc-KMT-proficient HAP1 cells (WT and KO cells reconstituted with WT ATPSc-KMT) or ATPSc-KMT-deficient HAP1 cells (KO and KO cells reconstituted with ATPSc-KMT E117A mutant) (n=9). **C)** Course of $PGE_2$-induced thermal hyperalgesia after intrathecal injection of mito-tempol (25 ug)) in mice expressing ATPSc-KMT in DRG neurons. Intraplantar HSV injections were administrated at day -3 and -1 (35.000 pfu/paw). **D)** Example pictures of ATF3 fluorescence in DRG neurons of non-primed and primed mice (6 hours after $PGE_2$ injection). Data are represented as mean ± SD. *P < 0.05, **P < 0.01, ***P < 0.001. Statistical analyses were performed by one-way ANOVA (B) followed by Dunnett's multiple comparison test or two-way repeated measures ANOVA followed by a post-hoc Sidak's multiple comparison test (A and C).
